# Dynamic frontotemporal systems process space and time in working memory

**DOI:** 10.1371/journal.pbio.2004274

**Published:** 2018-03-30

**Authors:** Elizabeth L. Johnson, Jenna N. Adams, Anne-Kristin Solbakk, Tor Endestad, Pål G. Larsson, Jugoslav Ivanovic, Torstein R. Meling, Jack J. Lin, Robert T. Knight

**Affiliations:** 1 Helen Wills Neuroscience Institute, University of California, Berkeley, Berkeley, California, United States of America; 2 Department of Psychology, University of California, Berkeley, Berkeley, California, United States of America; 3 Department of Psychology, Faculty of Social Sciences, University of Oslo, Oslo, Norway; 4 Department of Neurosurgery, Division of Clinical Neuroscience, Oslo University Hospital, Rikshospitalet, Oslo, Norway; 5 Department of Neuropsychology, Helgeland Hospital, Mosjøen, Norway; 6 Institute of Clinical Medicine, Faculty of Medicine, University of Oslo, Oslo, Norway; 7 Comprehensive Epilepsy Program, Department of Neurology, University of California, Irvine, Irvine, California, United States of America; University of Oxford, United Kingdom of Great Britain and Northern Ireland

## Abstract

How do we rapidly process incoming streams of information in working memory, a cognitive mechanism central to human behavior? Dominant views of working memory focus on the prefrontal cortex (PFC), but human hippocampal recordings provide a neurophysiological signature distinct from the PFC. Are these regions independent, or do they interact in the service of working memory? We addressed this core issue in behavior by recording directly from frontotemporal sites in humans performing a visuospatial working memory task that operationalizes the types of identity and spatiotemporal information we encounter every day. Theta band oscillations drove bidirectional interactions between the PFC and medial temporal lobe (MTL; including the hippocampus). MTL theta oscillations directed the PFC preferentially during the processing of spatiotemporal information, while PFC theta oscillations directed the MTL for all types of information being processed in working memory. These findings reveal an MTL theta mechanism for processing space and time and a domain-general PFC theta mechanism, providing evidence that rapid, dynamic MTL–PFC interactions underlie working memory for everyday experiences.

## Author summary

How do we rapidly process incoming streams of information in working memory? Dominant views of working memory focus on the prefrontal cortex (PFC), but other data suggest a role for the medial temporal lobe (MTL). To delineate whether (and how) these brain regions interact during working memory, we recorded directly from PFC and MTL sites in humans performing a task that tests working memory for the types of “what,” “where,” and “when” information encountered every day. MTL oscillations in the theta band (3–7 Hz) directed PFC activity during the processing of spatiotemporal information, while PFC theta oscillations directed MTL activity for all types of information. This functional dissociation provides the first demonstration of bidirectional communication between the PFC and MTL during working memory. Our findings reveal that rapid, dynamic interactions between these two regions underlie working memory for everyday experiences, challenging dominant views on the central role of the PFC.

## Introduction

The ability to hold and manipulate features of information in working memory provides the neurobiological infrastructure for our cognitive experiences. For 80 y, dominant views of working memory have focused on the prefrontal cortex (PFC) [1]. However, direct recordings from the human hippocampus—best known for its pivotal role in long-term memory [2]—have identified a neurophysiological signature of working memory that is distinct from the PFC [3–4]. Furthermore, the hippocampus and surrounding medial temporal structures (together, the MTL) track performance on tasks that vary the amount of information being represented [5] and are coactive with the PFC on more demanding tasks [6–7]. Recent proposals also suggest that the MTL is critical to working memory for spatiotemporal context, as it is for long-term memory [8–10]. These findings raise the question of how the MTL and PFC interact during working memory or, rather, whether the MTL and PFC contribute independently to working memory. Using a task that operationalizes identity and spatiotemporal information, we show that theta oscillations recorded directly from the human brain drive simultaneous, bidirectional MTL–PFC interactions. Our findings provide evidence for bidirectional MTL–PFC communication in humans (see [11–13] for evidence from animal physiology) and suggest that dynamic MTL–PFC interactions underlie working memory for everyday experiences.

**Fig 1:**
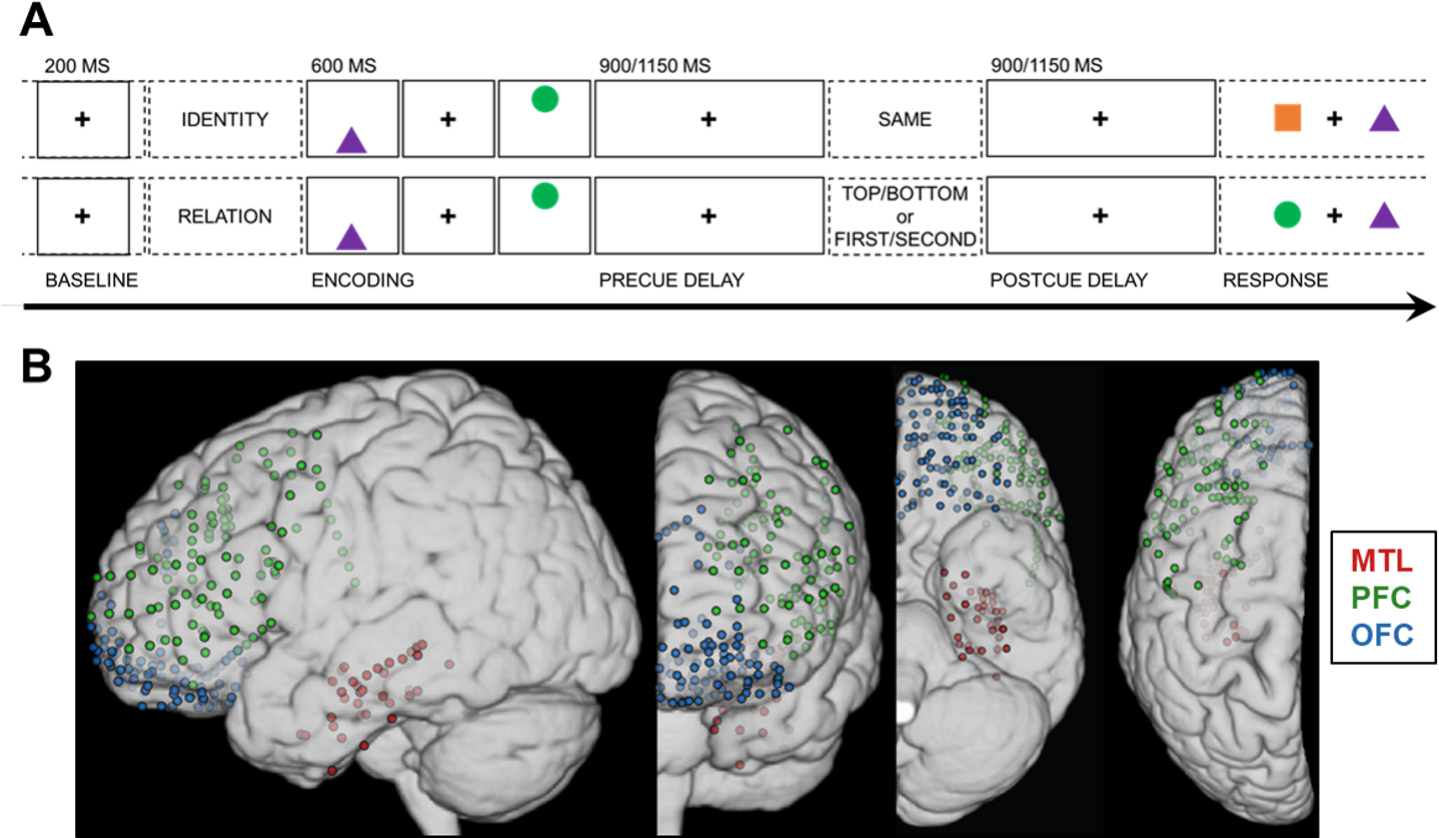
Working memory task design and electrode coverage. (A) Single-trial working memory task design. Following a 1-s pretrial fixation interval (−250 to −50 ms pretrial epoch used as baseline), subjects were directed to focus on either “identity” or “relation” information. Then, 2 common shapes were presented for 200 ms each in a specific spatiotemporal configuration (i.e., top/bottom spatial and first/second temporal positions). After a 900- or 1,150-ms jittered precue fixation delay, the test cue appeared (i.e., one word presented on screen for 800 ms), followed by a postcue fixation delay of the same length. Working memory was tested in a two-alternative forced choice test (0.5 chance rate). In the identity test (top), subjects indicated whether the pair was the “same” pair they just studied (correct response in this example: “no”). In the spatiotemporal relation test (bottom), subjects indicated which shape fit the top/bottom spatial or first/second temporal relation cue (correct response for cue “top” or “second”: “circle”). (B) Reconstruction of electrode coverage for all subjects. Electrodes are overlaid on the left hemisphere, displayed in 4 views. Red = MTL; green = PFC; blue = OFC. Underlying data can be found in University of California, Berkeley, Collaborative Research in Computational Neuroscience database (http://dx.doi.org/10.6080/K0VX0DQD). MTL, medial temporal lobe; OFC, orbitofrontal cortex; PFC, prefrontal cortex.

Intracranial electrophysiology provides rare access to both neocortical and subcortical structures in humans with millisecond precision, offering unparalleled insight into our neurocognitive experiences [14]. We recorded directly from frontal and temporal sites in 10 adults (mean ± SD [range]: 37 ± 13 [22–69] y of age) while they performed a visuospatial working memory task that is known to recruit the lateral PFC [15]. Each trial consisted of 5 phases: pretrial, encoding, precue delay, postcue delay, and response (Fig 1A). Following pretrial central fixation, 2 common shapes were presented sequentially in a top/bottom spatial orientation. Then, a test cue was presented mid-delay to retroactively cue specific information about the items being maintained in working memory [16]: “same” (identities; Fig 1A top panel), “top/bottom” (spatial relations, bottom panel), or “first/second” (temporal relations, bottom panel). This critical manipulation allowed us to examine how working memory unfolded over time, with a focus on the selection of identity, spatial, or temporal information during the postcue delay. We first analyzed the encoding and delay intervals for correct-response trials relative to the pretrial baseline. We then investigated working memory for space and time by comparing activity during the selection of an item in space or time to the ongoing maintenance of item identity. Because the maintenance of information about the identity of the shapes was common to all conditions [8], identity trials provided a model control condition against which to contrast working memory for spatial and temporal information.

Cross-frequency coupling between the phase of slow oscillations (e.g., theta band) and amplitude of faster oscillations (e.g., broadband gamma) is posited to play an important role in neuronal computation and communication. Indeed, phase-amplitude coupling (PAC) has been observed locally and interregionally across a wide range of cognitive tasks in humans and animals [3–4,14,17–24] (see also [25] for evidence of phase-neuronal coupling in humans). PAC provides a potential mechanism for information transfer between large-scale networks operating at subsecond, behavioral timescales and the local, fast activity of neuronal populations [18–19]. During human working memory, hippocampal PAC between theta oscillations and broadband beta–gamma activity predicts individual memory performance and varies according to the amount of information being represented, uncovering PAC as a neurophysiological signature of multi-item working memory [3–4]. We hypothesized that the MTL and PFC would interact to support working memory via PAC. Specifically, dynamic fluctuations in PAC would provide a mechanism for the online communication of information about item identity and spatiotemporal context. Our hypothesis builds on recent evidence that fluctuations in PAC predict population-level spiking patterns [20], making it an ideal metric for tracking rapid, behaviorally relevant shifts in neuronal population activity. Furthermore, by taking this mechanistic approach, we aimed to investigate whether the MTL or PFC would direct frontotemporal interactions in the service of working memory.

## Results

Signals were recorded directly from MTL (*n* = 36 electrodes after bipolar montage rereferencing), lateral PFC (*n =* 116), and orbitofrontal cortex (OFC; *n =* 102) sites (Fig 1B). Due to the nature of the recording technique, the electrode arrays covered the MTL and PFC within the same hemisphere in 9 out of 10 subjects and the MTL and OFC in 7 out of 10 subjects. This rich dataset allowed us to investigate each region independently, as well as test for directional interactions between MTL and frontal regions. The inclusion of OFC electrodes provided an additional opportunity to compare the effects observed in this region—which is anatomically connected to the MTL—to the effects observed in the MTL and PFC.

We first confirmed that all subjects were proficient at the task (accuracy range 0.79–0.97, chance 0.5) and that any condition effects observed in the neural data could not be attributed to difficulty (mixed-effects model, accuracy: F_1,28_ < 0.10, *p >* 0.75; correct-trial response time: F_1,28_ < 2.55, *p >* 0.12; mean ± SD, identity: 0.90 ± 0.04, 1659 ± 701 ms; spatial relation: 0.92 ± 0.09, 1335 ± 574 ms; temporal relation: 0.89 ± 0.09, 1475 ± 734 ms). We then submitted all 254 electrodes to analyses of task-induced event-related potentials (ERPs), spectrotemporal power, and interelectrode connectivity and took a step-wise, data-driven approach to analyze PAC. Finally, we submitted the PAC data outputs to group-level statistical models, both by region (i.e., MTL, PFC, OFC) and interregionally (MTL–PFC, MTL–OFC), to isolate the system supporting working memory for space and time.

### Theta activity is sustained during encoding and delay

We first quantified ERPs (1–30 Hz bandpass) over the 200-ms pretrial baseline, 1,500-ms encoding and precue delay interval, and 900-ms postcue delay for correct trials (see Fig 1A) [15]. Then, encoding and delay outputs were absolute baseline-corrected on the temporal mean of the pretrial baseline. Cluster-based permutation testing [26] indicated that ERPs did not differ between identity, spatial, and temporal conditions in any electrode (cluster-corrected *p >* 0.05; S1 Fig), ensuring that any spectral or cross-spectral condition effects were not due to exogenous activity measured in ERPs.

**Fig 2:**
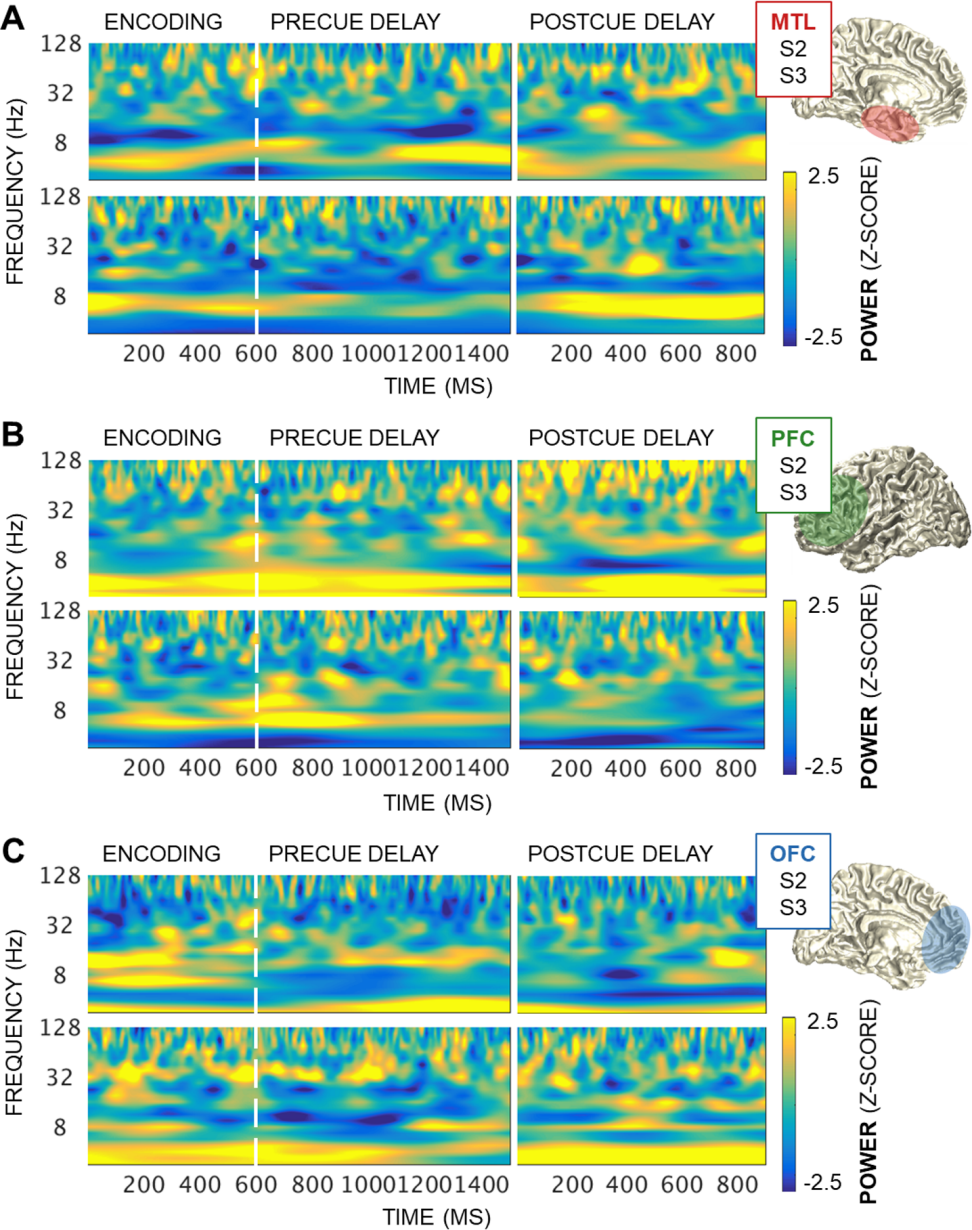
Task-induced power at encoding and delay. (A) Task-induced power over encoding, precue, and postcue intervals in representative MTL electrodes from 2 Ss. The MTL showed sustained theta band (3–7 Hz) activity, narrowband alpha desynchronization, and variable, transient activities above 16 Hz (|z| > 1.96, *p <* 0.05). Anatomy, S2: CA1 (hippocampus); S3: perirhinal cortex. (B) Equivalent to panel A: the PFC showed sustained theta band activity and variable, transient activities above 9.5 Hz (|z| > 1.96, *p <* 0.05). Anatomy, S2 and S3: middle/superior frontal gyrus (dorsolateral PFC). (C) Equivalent to panel A: the OFC showed sustained theta band activity, periodic narrowband alpha, and variable, transient activities above 16 Hz (|z| > 1.96, *p <* 0.05). Anatomy, S2 and S3: medial OFC. Underlying data can be found in University of California, Berkeley, Collaborative Research in Computational Neuroscience database (http://dx.doi.org/10.6080/K0VX0DQD). MTL, medial temporal lobe; OFC, orbitofrontal cortex; PFC, prefrontal cortex; S, subject.

We then proceeded to examine spectral components. Analysis of task-induced power identified widespread, sustained oscillatory activity centered in the theta band (3–7 Hz) in frontotemporal regions (Fig 2). The pretrial baseline, encoding and precue, and postcue data segments were zero padded to 7,500 ms, bandpass filtered between 1 and 200 Hz at 24 logarithmically spaced frequencies, and Hilbert transformed. Analytic amplitude envelopes were extracted and squared to compute power, and then encoding and delay outputs were standardized on the pretrial baseline. The raw power outputs were z-scored against pretrial baseline distributions generated by randomly selecting baseline data samples to assess the significance of task-induced power effects per trial (i.e., statistical bootstrapping) [15]. Single-subject analyses revealed sustained theta band (centered at 3–7 Hz) and variable transient activities at higher frequencies (>16 Hz) in all regions (z > 1.96, *p <* 0.05; Fig 2). Activity in the alpha band (centered at 8–13 Hz) was desynchronized in the MTL (z < −1.96, *p <* 0.05) and less sustained in the PFC than the OFC, highlighting theta as the most prominent slow oscillation across frontotemporal regions in this working memory task.

Cluster-based permutation testing indicated that power did not differ between identity, spatial, and temporal conditions at any time–frequency point in 246 out of 254 electrodes (cluster-corrected *p >* 0.05). Because condition differences in power at the input data frequencies can create spurious PAC effects [27], the 8 electrodes exhibiting power effects (5 PFC plus 3 OFC across 4 out of 10 subjects) were excluded from further analysis.

### Theta oscillations direct local and long-range activity during information processing in working memory

**Fig 3:**
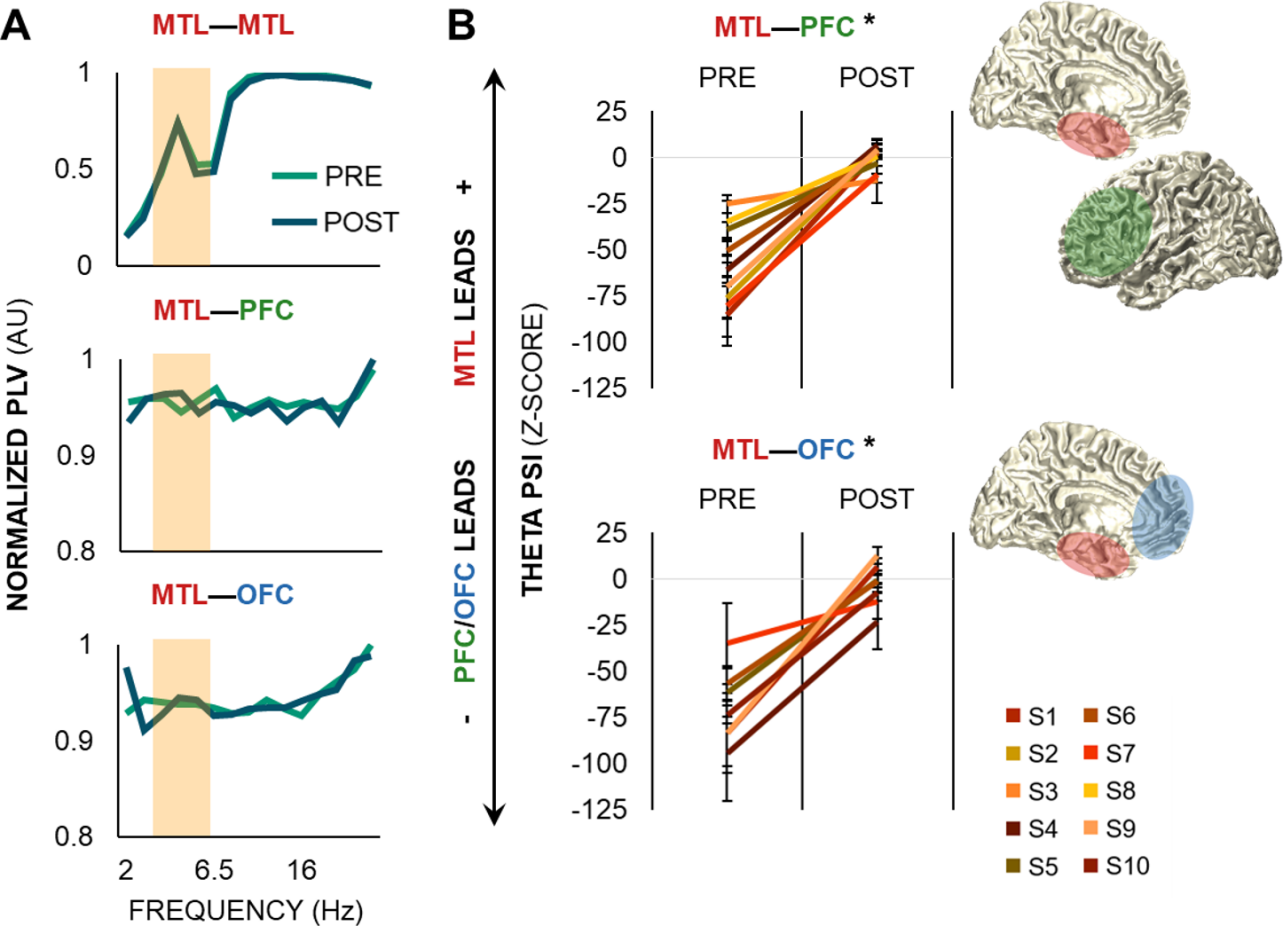
Theta oscillations link frontotemporal regions during working memory. (A) PLVs over pre- and postcue delays within all MTL electrodes (top) and between all MTL and PFC (middle) and OFC (bottom) electrodes in a representative S. Theta and alpha peaks were observed within the MTL and between MTL and frontal regions over delay. Data are represented as the temporal mean of each delay interval, normalized by the maximum PLV across all electrodes. The shaded area indicates the theta range used for PSI analysis, as depicted in panel B. (B) Theta band PSI shifted from a unidirectional, frontal-driven network during the precue delay to a bidirectional, MTL-frontal network during the postcue delay (*p <* 4 × 10^−27^). Data are represented as mean ± SEM per S; positive values indicate that the MTL leads the PFC (top) or OFC (bottom), and negative values indicate that the PFC/OFC leads the MTL. * = significant effect. Underlying data can be found in University of California, Berkeley, Collaborative Research in Computational Neuroscience database (http://dx.doi.org/10.6080/K0VX0DQD). MTL, medial temporal lobe; OFC, orbitofrontal cortex; PFC, prefrontal cortex; PLV, phase-locking value; POST, postcue delay; PRE, precue delay; PSI, Phase Slope Index; S, subject.

Frontotemporal regions were synchronized across multiple frequencies during the delay intervals, with narrowband oscillatory peaks detected in the theta range that shifted in direction upon presentation of the critical test cue. First, we computed phase-locking values (PLVs) up to 20 Hz from the imaginary part of the Hilbert-transformed complex signal, minimizing the potential influence of volume conduction [28–29]. This analysis confirmed peak theta PLVs within the MTL and between MTL and frontal sites, as well as more broadband synchrony in the alpha range (Fig 3A). We then used the Phase Slope Index (PSI) to quantify theta band directional connectivity between MTL and frontal regions [30], as well as cross-spectral directionality between theta oscillations and all higher-frequency amplitude envelopes within and between MTL and frontal regions [21]. The PSI metric tracks whether the slope of the phase lag between A–B signal pairs is consistent across several adjacent frequency bins; positive PSI indicates that signal A leads signal B, negative PSI indicates the reverse, and zero PSI indicates either zero or an evenly balanced lead–lag relationship between signals. We chose to use PSI for consistency across analyses of theta band and cross-spectral directionality because Granger-based approaches are especially sensitive to signal-to-noise ratio [31–32], making them poorly suited for assessing different spectral signals, which often differ in signal-to-noise ratio.

The 900-ms pre- and postcue delay data segments for all correct trials were zero padded to 4 s and multiplied with a Hanning taper from 3 and 7 Hz (1-Hz steps, 2-Hz bandwidth), from which we computed theta band PSI [30]. Raw PSI outputs were z-scored against null distributions generated by randomly shuffling the frequency bins to correct for any spurious results and assess the significance of directional effects [15,31]. The precue delay was characterized by unidirectional frontal-to-MTL theta band connectivity (threshold z < −1.96, *p <* 0.05), consistent with dominant models attributing working memory maintenance to frontal control over posterior regions [1]. However, following presentation of the test cue—which cued the subject to prioritize and select identity, spatial, or temporal information in working memory (see Fig 1A)—directionality shifted between frontotemporal regions from a unidirectional to bidirectional network (postcue > precue, MTL–PFC: F_1,342_ > 139, *p <* 4 × 10^−27^; MTL–OFC: F_1,604_ > 268, *p <* 4 × 10^−50^; Fig 3B). Within individual subjects, we observed that some electrodes shifted in directionality from frontal to MTL leads, while others simply decreased in the strength of frontal lead over the MTL, reflecting spatially diverse patterns of bidirectional frontotemporal theta connectivity during information processing.

Importantly, analysis of cross-spectral directionality revealed that working memory demands also shifted cross-spectral oscillatory activity following presentation of the test cue. To quantify cross-spectral directionality, we separately extracted the higher-frequency analytic amplitude envelopes (from alpha through high-frequency broadband ranges) using the Hilbert bandpass technique and then passed each frequency-range amplitude output through the same 3- to 7-Hz spectral decomposition procedure as in theta band PSI. PSI was quantified between the actual theta signal and each amplitude frequency range [21], and then the raw outputs were z-scored against null distributions generated by randomly shuffling the amplitude frequency bins [15,31]. Finally, outputs were averaged across frequencies to index which intraregional electrodes (i.e., MTL, PFC, OFC) and interregional, within-hemisphere electrode pairs (e.g., left MTL–PFC and PFC–MTL) exhibited theta phase directionality to higher-frequency amplitudes.

**Fig 4:**
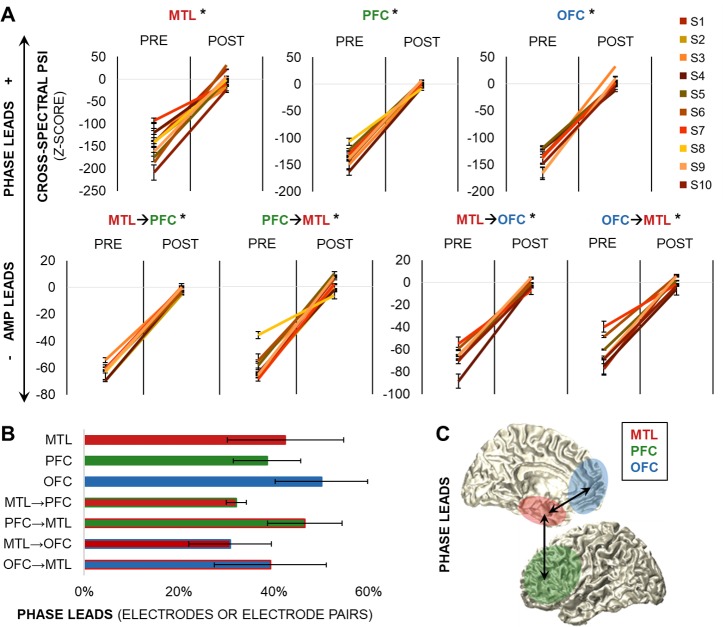
Theta oscillations direct cross-spectral activity during information processing. (A) Theta phase–amplitude directionality shifted from pre- to postcue delay both locally and across regions (*p <* 7 × 10^−26^). Data are represented as mean ± SEM per S; positive values indicate that phase leads amplitude, and negative values indicate that amplitude leads phase. * = significant effect. (B) Percentage of individual electrodes and electrode pairs in which theta phase directed higher-frequency amplitudes during the postcue delay. Data are represented as mean ± SEM. (C) Schematic of theta phase–led bidirectional frontotemporal interactions during the postcue delay. Underlying data can be found in University of California, Berkeley, Collaborative Research in Computational Neuroscience database (http://dx.doi.org/10.6080/K0VX0DQD). AMP, amplitude; MTL, medial temporal lobe; OFC, orbitofrontal cortex; PFC, prefrontal cortex; POST, postcue delay; PRE, precue delay; S, subject.

None of the electrodes or interregional electrode pairs were identified as significantly theta phase directing during the precue delay—which indicates that theta oscillations did not modulate higher-frequency activity and precludes further interpretation of theta-driven PAC during the precue delay [21]. However, following presentation of the test cue, cross-spectral directionality shifted within and between all frontotemporal regions to a theta phase–driven network during the postcue delay (postcue > precue, MTL: F_1,70_ > 273, *p <* 7 × 10^−26^; PFC: F_1,220_ > 1 × 10^3^, *p <* 2 × 10^−88^; OFC: F_1,196_ > 911, *p <* 2 × 10^−75^; MTL→PFC: F_1,342_ > 1 × 10^3^, *p <* 7 × 10^−129^; PFC→MTL: F_1,342_ > 1 × 10^3^, *p <* 4 × 10^−121^; MTL→OFC: F_1,604_ > 2×10^3^, *p <* 8 × 10^−211^; OFC→MTL: F_1,604_ > 2 × 10^3^, *p <* 4 × 10^−208^; Fig 4A). Significant theta phase leads over amplitude were observed in a subset of all individual electrodes and electrode pairs (threshold z > 1.96, *p <* 0.05; Fig 4B). Taking suprathreshold phase directionality as the criterion for electrode selection (see [22] for a comparable approach), we submitted only the electrodes and electrode pairs that showed significant theta phase leads over amplitude to further analysis of cross-spectral directionality and PAC (*n =* 13 MTL, 52 PFC, 38 OFC, 56 MTL→PFC, 85 PFC→MTL, 93 MTL→OFC, and 115 OFC→MTL).

Importantly, the observation of theta phase directionality in both MTL→PFC and PFC→MTL directions, as well as MTL→OFC and OFC→MTL directions, provided corroborating evidence for parallel, bidirectional frontotemporal interactions during information processing in working memory (Fig 4C). We used linear mixed-effects models to confirm that the strength of theta phase directionality was equal across the amplitude frequency ranges (alpha through high-frequency broadband) and in both MTL→PFC/OFC and PFC/OFC→MTL directions (uncorrected *p >* 0.11; S1 Table).

### Dynamic theta PAC tracks information in working memory

**Fig 5:**
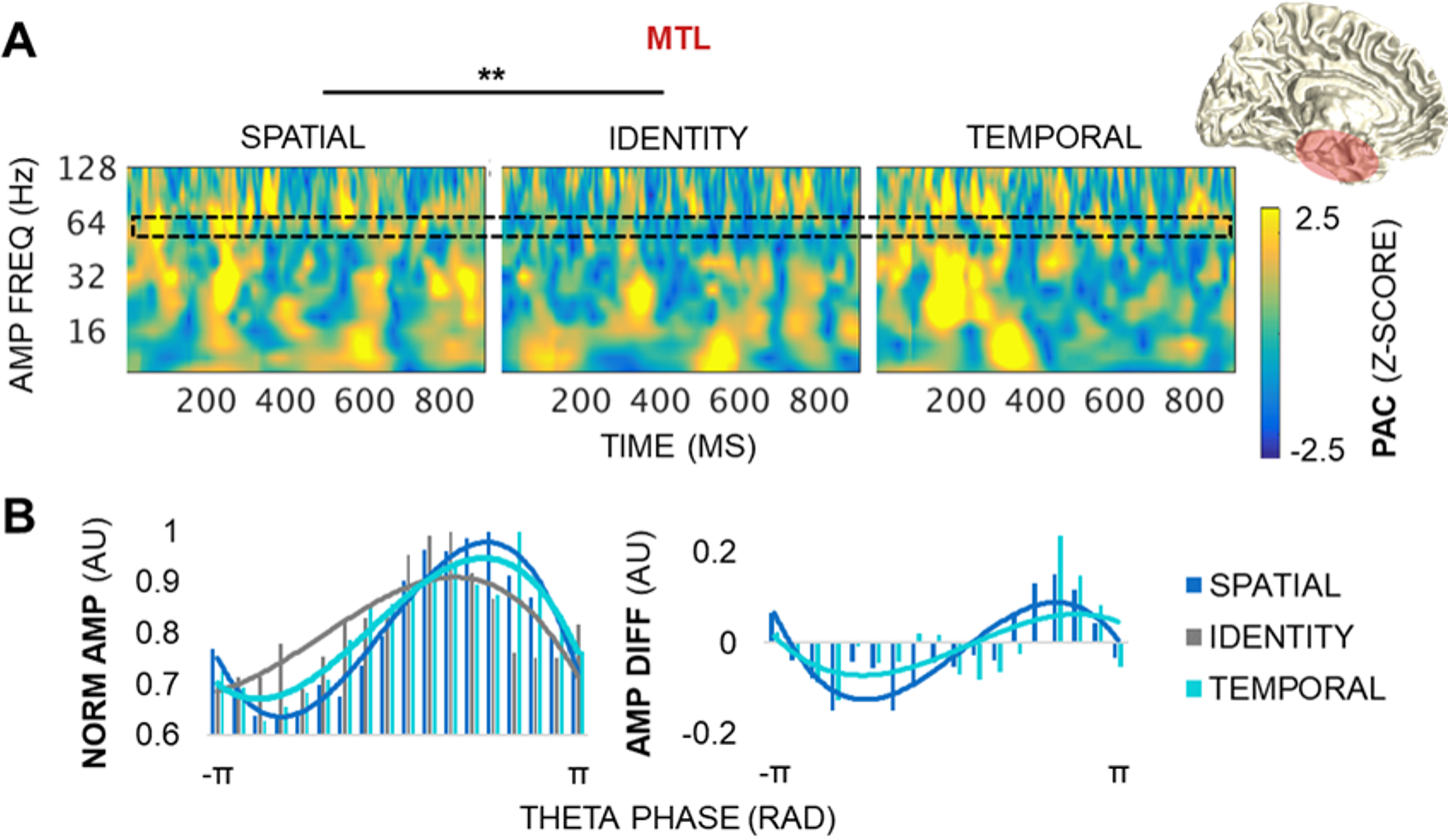
MTL theta PAC dynamics track space. (A) PAC by condition during the postcue delay in a representative MTL electrode. The MTL showed variable, transient PAC across the spectrum of amplitudes (z > 1.96, *p <* 0.05). PAC was greater for spatial than identity information, with peak differences at 22.5- and 64-Hz amplitudes (*p <* 3 × 10^−5^). The black block indicates the amplitude data range depicted in panel B. ** = significant condition and condition × frequency effects. (B) The distribution of raw higher-frequency amplitudes across 18 theta phase bins, by condition, normalized by the maximum amplitude across all of the phase bins. AMP frequency range: broadband gamma (centered at 64 Hz). Underlying data can be found in University of California, Berkeley, Collaborative Research in Computational Neuroscience database (http://dx.doi.org/10.6080/K0VX0DQD). AMP, amplitude; DIFF, difference (i.e., spatial–identity, temporal–identity); FREQ, frequency; MTL, medial temporal lobe; NORM, normalized; PAC, phase-amplitude coupling; RAD, radians.

**Fig 6:**
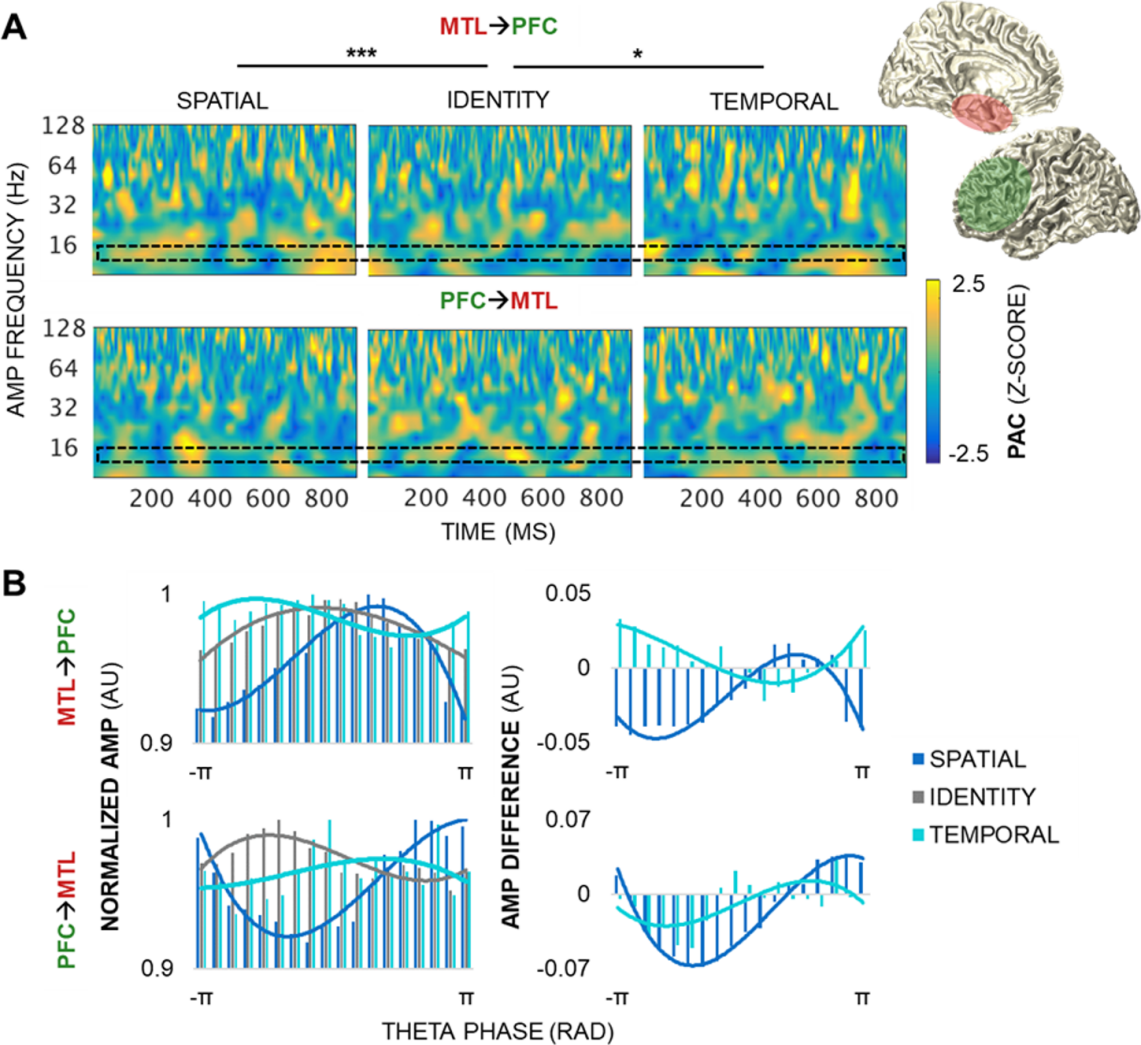
MTL→PFC theta PAC dynamics track both space and time. (A) PAC by condition during the postcue delay in a representative MTL–PFC electrode pair. MTL**→**PFC (top) and PFC**→**MTL (bottom) pairs showed variable, transient PAC across the spectrum of amplitudes (z > 1.96, *p <* 0.05). MTL**→**PFC PAC was greater for spatial than identity information, with peak differences at 13.5- to 16-Hz amplitudes during the 100- to 250- and 800- to 900-ms epochs (*p <* 2 × 10^−4^). MTL**→**PFC PAC was also greater for temporal than identity information (*p <* 9 × 10^−5^). No condition differences were observed in the PFC**→**MTL direction. The black block indicates the amplitude data range depicted in panel B. * = significant condition effect; *** = significant condition, condition × frequency, condition × time, and 3-way interaction effects. (B) The distribution of raw higher-frequency amplitudes across 18 theta phase bins, by condition, normalized by the maximum amplitude across all of the phase bins. Underlying data can be found in University of California, Berkeley, Collaborative Research in Computational Neuroscience database (http://dx.doi.org/10.6080/K0VX0DQD). AMP frequency range: beta (centered at 13.5 Hz). AMP, amplitude; MTL, medial temporal lobe; PAC, phase-amplitude coupling; PFC, prefrontal cortex; RAD, radians.

**Fig 7:**
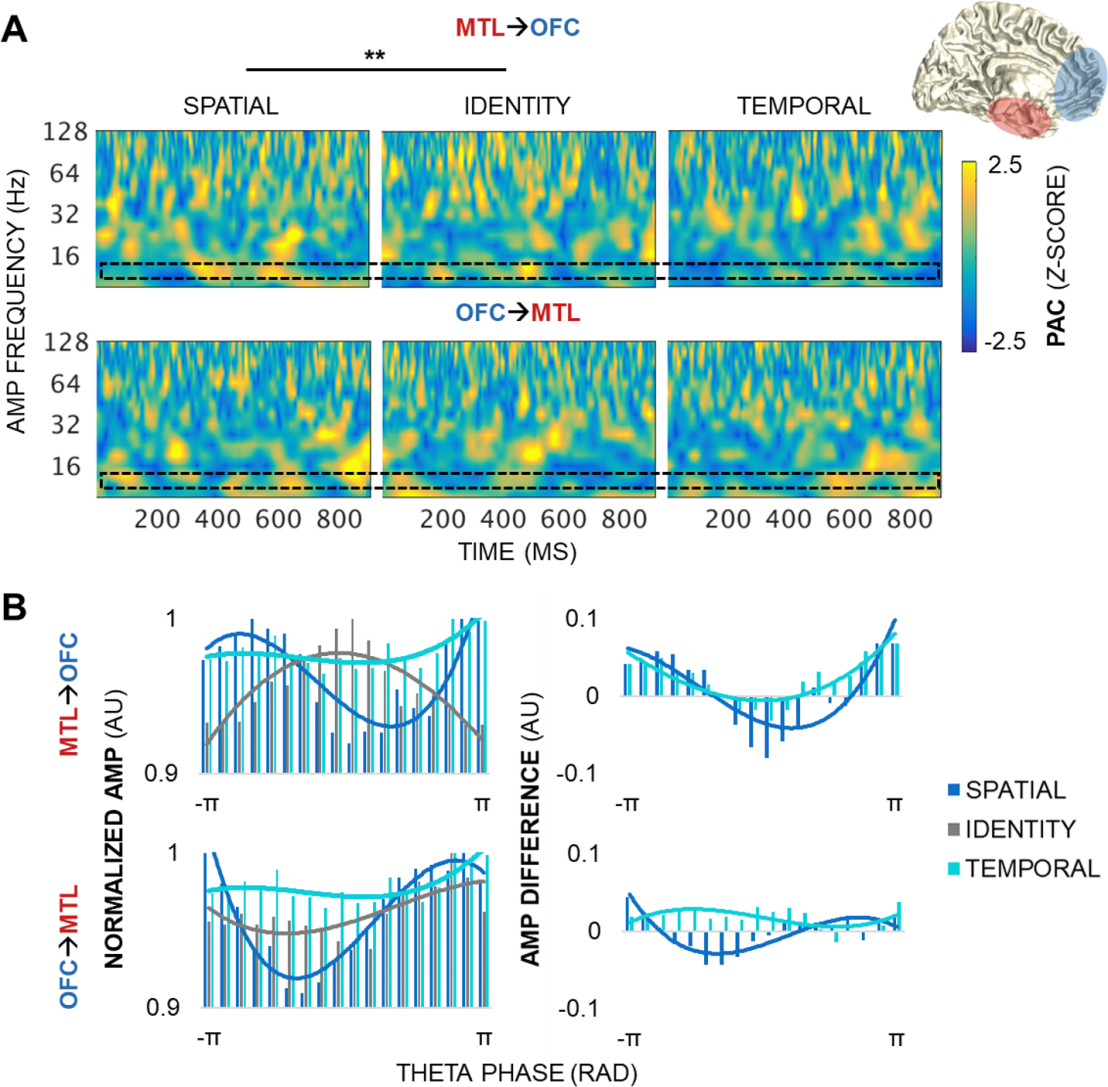
MTL→OFC theta PAC dynamics track space but not time. (A) PAC by condition during the postcue delay in a representative MTL–OFC electrode pair. MTL**→**OFC (top) and OFC**→**MTL (bottom) pairs showed variable, transient PAC across the spectrum of amplitudes (z > 1.96, *p <* 0.05). MTL**→**OFC PAC was greater for spatial than identity information, with peak differences at 9.5-Hz amplitudes (*p <* 5 × 10^−3^). No condition differences were observed in the OFC**→**MTL direction. The black block indicates the amplitude data range depicted in panel B. ** = significant condition and condition × frequency effects. (B) The distribution of raw higher-frequency amplitudes across 18 theta phase bins, by condition, normalized by the maximum amplitude across all of the phase bins. AMP frequency range: alpha (centered at 9.5 Hz). Underlying data can be found in University of California, Berkeley, Collaborative Research in Computational Neuroscience database (http://dx.doi.org/10.6080/K0VX0DQD). AMP, amplitude; MTL, medial temporal lobe; OFC, orbitofrontal cortex; PAC, phase-amplitude coupling; RAD, radians.

Analysis of spectrotemporal PAC identified dynamic fluctuations in PAC throughout frontotemporal networks during the selection of identity, spatial, and temporal information (Figs 5–7, S2). Instantaneous theta phase (at individually determined frequencies centered at 3.5–6.5 Hz, 3-Hz bandwidth) and higher-frequency analytic amplitude envelopes were extracted using the Hilbert bandpass technique, from which spectrotemporal PAC was quantified using circular statistics [22–23]. The Pearson correlation was calculated between theta phase and each amplitude frequency point across correct trials within each condition, and then raw PAC outputs were z-scored against null distributions generated by randomly shuffling the amplitude frequencies across trials to correct for any spurious results and assess the significance of PAC effects [23,27,31]. PAC was visualized per electrode and directional electrode pair, which revealed dynamic fluctuations in PAC across the spectrum of amplitudes in all frontotemporal electrodes and directional electrode pairs (z > 1.96, *p <* 0.05; Figs 5–7, S2). These results provide evidence that frontotemporal theta oscillations rapidly and flexibly coordinate activity—both locally and across long-range networks [22,24]—during the selection of different types of information. Furthermore, the detection of PAC throughout frontotemporal regions reveals that the previously reported hippocampal signature of working memory is a dynamic, network-level phenomenon.

We submitted the spectrotemporal PAC data to a series of group linear mixed-effects models to test our hypothesis that dynamic fluctuations in PAC would define distinct network patterns for information being represented in working memory. PAC during the selection of an item in space or time was compared to that for the ongoing maintenance of item identity (see Fig 1A). PAC data were submitted to separate models by regional (i.e., MTL, PFC, OFC) and directional system (MTL**→**PFC, PFC**→**MTL, MTL**→**OFC, OFC**→**MTL) with condition-, amplitude frequency–, and time-fixed effects, and electrode/electrode pair–and subject-random effects. The results of these models isolate which frontotemporal systems exhibit dynamic PAC effects that track working memory for space and/or time.

Locally within the MTL, PAC was greater for spatial than identity information (condition *p <* 9 × 10^−6^), with peak differences at broadband beta and gamma amplitudes (centered at 22.5 and 64 Hz; condition × frequency *p <* 3 × 10^−5^; Fig 5, S2 Table). No differences were observed in the temporal information model (i.e., temporal versus identity information; Bonferroni-corrected *p >* 0.05). In contrast, no condition effects were observed within the PFC or OFC (Bonferroni-corrected *p >* 0.05; S2 Fig, S2 Table), indicating that frontal PAC did not distinguish between identity, spatial, and temporal information in working memory. Because the PFC and OFC processed information about spatiotemporal relationships between stimuli along with that about stimulus identity, these results suggest a domain-general role for the frontal cortex in working memory.

Between regions, PAC was greater for spatial than identity information in the MTL**→**PFC direction (condition *p <* 2 × 10^−10^), with peak differences at PFC beta amplitudes (centered at 13.5–16 Hz) during the 100- to 250-ms and 800- to 900-ms epochs (condition × frequency *p <* 2 × 10^−4^; condition × time *p <* 2×10^−9^; condition × frequency × time *p* = 4 × 10^−4^; Fig 6 top, S3 Table). MTL**→**PFC PAC was also greater for temporal than identity information (condition *p <* 9 × 10^−5^). In contrast, no condition effects were observed in the PFC**→**MTL direction (Bonferroni-corrected *p >* 0.05; Fig 6 bottom, S3 Table). In the MTL**→**OFC direction, PAC was greater for spatial than identity information (condition *p <* 4 × 10^−6^), with peak differences at alpha amplitudes (centered at 9.5 Hz; condition × frequency *p <* 5 × 10^−3^; Fig 7 top, S4 Table). No effects were observed in the temporal information model or in the OFC**→**MTL direction (Bonferroni-corrected *p >* 0.05; Fig 7 bottom, S4 Table).

These results reveal that MTL theta oscillations direct dynamic PAC fluctuations locally in the MTL, and long-range in the PFC and OFC preferentially during the processing of spatial information, and only in the PFC during the processing of temporal information—supporting our hypothesis. Importantly, we observed that the MTL**→**PFC system was the only system that tracked working memory for both space and time. This finding demonstrates that working memory for spatiotemporal information is contingent on long-range, directional interaction from the MTL to the PFC. In contrast, PFC**→**MTL and OFC**→**MTL systems appear to serve a domain-general role, like local PFC and OFC systems, where information about spatiotemporal relationships between stimuli is processed in parallel to that about stimulus identity.

**Fig 8:**
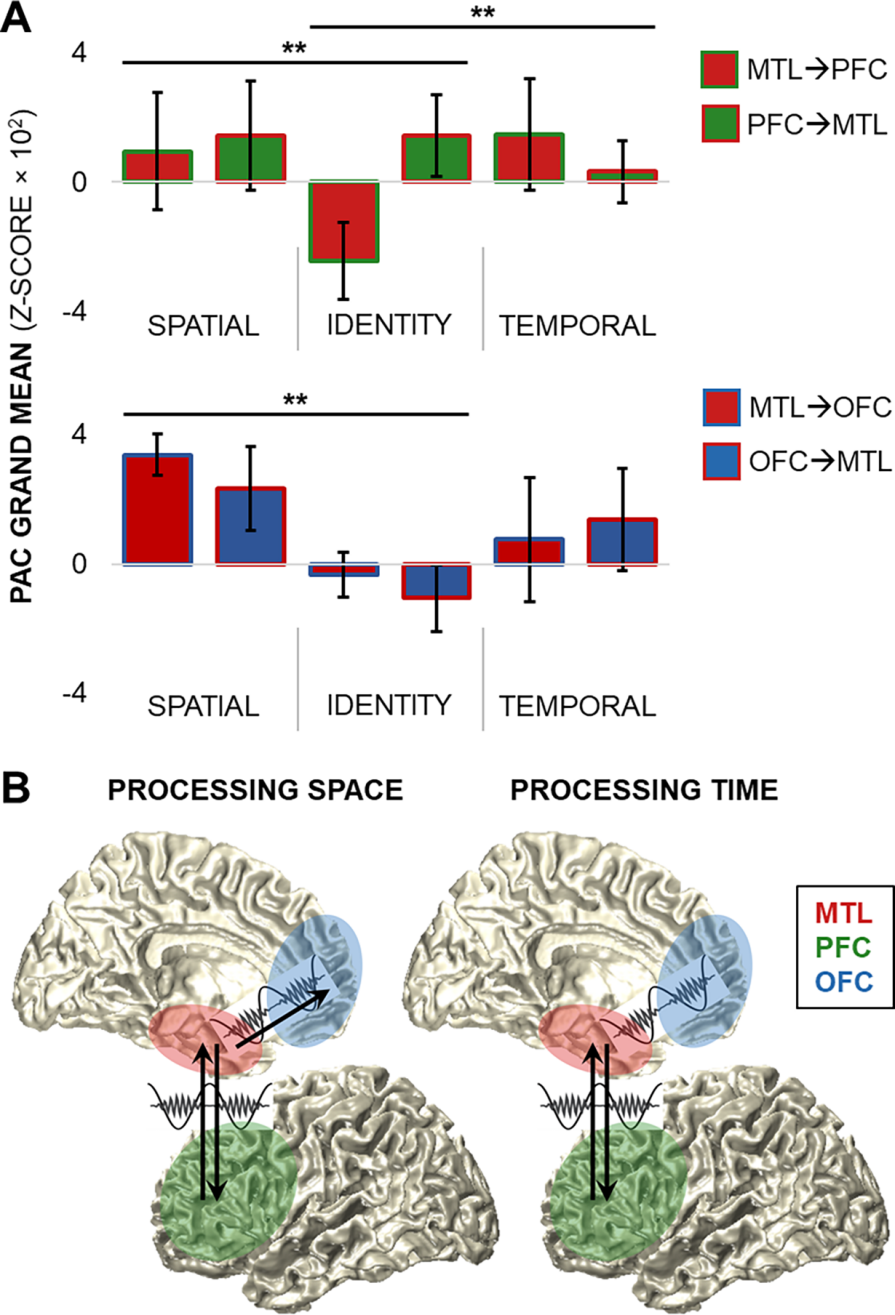
Bidirectional MTL–PFC theta networks for working memory. (A) PAC grand means by condition and direction for MTL–PFC (top) and MTL–OFC (bottom) networks. Condition moderated PAC in the MTL**→**PFC direction so that MTL**→**PFC PAC was greater for spatiotemporal than identity information (*p <* 2 × 10^−5^), while PFC**→**MTL PAC was greater than MTL**→**PFC PAC overall (*p <* 2 × 10^−9^), revealing bidirectional PAC for processing space and time. In contrast, MTL**→**OFC PAC was greater than OFC**→**MTL PAC, which was moderated by condition so that MTL**→**OFC PAC was greatest for spatial information. Data are represented as mean ± SEM. ** = significant direction and condition × direction effects. (B) Schematics of spatial (left) and temporal (right) information processing. The bidirectional MTL–PFC network subserves spatial and temporal information processing, while a relatively unidirectional MTL**→**OFC network is also involved in spatial information processing. Underlying data can be found in University of California, Berkeley, Collaborative Research in Computational Neuroscience database (http://dx.doi.org/10.6080/K0VX0DQD). MTL, medial temporal lobe; OFC, orbitofrontal cortex; PAC, phase-amplitude coupling; PFC, prefrontal cortex.

We performed posthoc statistical testing of the long-range network PAC data to further investigate the directional influence of medial temporal versus frontal theta oscillations in working memory. PAC data in both directions (e.g., MTL**→**PFC and PFC**→**MTL) were submitted to group linear mixed-effects models with a fourth fixed effect to model the strength of PAC by direction. The MTL–PFC models confirmed that the MTL directed the PFC preferentially during the processing of spatiotemporal over identity information (condition × direction; spatial model: *p <* 3 × 10^−8^; temporal model: *p <* 2 × 10^−5^; Fig 8A top, S3 Table). However, while PFC**→**MTL PAC did not differ by condition, PAC was enhanced in the PFC**→**MTL relative to MTL**→**PFC direction (direction; spatial model: *p <* 2 × 10^−11^; temporal model: *p <* 2 × 10^−9^). These results provide further evidence for functionally dissociable MTL**→**PFC and PFC**→**MTL theta oscillatory networks and suggest that our working memory for everyday experiences is contingent on simultaneous, bidirectional MTL–PFC interactions (Fig 8B). The MTL–OFC models indicated that the pattern of bidirectional interactions was unique to the MTL–PFC network. PAC was greater in the MTL**→**OFC direction than in reverse and greater for spatial than identity information in the same direction (direction *p <* 2 × 10^−3^; condition × direction *p <* 3 × 10^−4^; Fig 8A bottom, S4 Table), highlighting a relatively unidirectional MTL**→**OFC network for processing spatial information. No effects were observed in the MTL–OFC temporal model (Bonferroni-corrected *p >* 0.05).

## Discussion

Central to our cognitive experiences is the ability to simultaneously process streams of relevant information in working memory. Our findings suggest that this impressive feat of rapid, parallel processing is subserved by spatially distributed, bidirectional oscillatory interactions between the MTL and PFC—as indexed by phase synchrony, directional connectivity, cross-spectral directionality, and PAC. In the current task, subjects encoded pairs of common shapes in specific spatiotemporal orientations, and a test cue was presented mid-delay so that we could isolate working memory for space and time by contrasting the selection of an item in space or time against the ongoing maintenance of item identity. First, analysis of task-induced power revealed sustained theta band oscillatory activity in all frontotemporal sites during the encoding and delay intervals of information that was to be remembered. Second, analyses of interelectrode phase synchrony and directional connectivity revealed task-induced frontotemporal interactions in the theta band as well as spatially distributed patterns of bidirectional interactions during the postcue delay. Third, analysis of cross-spectral directionality revealed that presentation of the test cue reset networks of local and long-range interactions so that theta oscillations directed higher-frequency activities throughout frontotemporal regions. These corroborating results evidence changes in theta band oscillatory and network-level patterns linked to updating the contents of working memory [16,33,34]. We then investigated the hypothesis that dynamic fluctuations in theta PAC, a signature of multi-item working memory in the human hippocampus [3–4], would support working memory for space and time.

Locally within the MTL, we found that theta PAC to broadband gamma activity (centered at 64 Hz) preferentially tracked working memory for an item in space over the ongoing maintenance of item identity. This result is consistent with a wealth of evidence for MTL theta-gamma PAC in human spatial navigation and memory [18–19] and theta-entrained neuronal activity in animal models [35], suggesting that humans recruit the well-established mammalian infrastructure for spatial navigation to process spatial information in working memory. In addition, we observed that MTL theta oscillations directed local MTL and long-range frontal activities in the alpha (centered at 9.5 Hz) and beta (13.5–22.5 Hz) bands preferentially during the processing of spatial information. These findings suggest that MTL theta oscillations may also direct multiple hierarchical networks [36]—operating at high-beta timescales locally in the MTL, low-beta timescales in the PFC, and alpha timescales in the OFC—to process spatial information. Because task-induced beta activity was transient in the MTL and PFC and alpha activity was more sustained in the OFC, we suggest that MTL-guided oscillatory systems for spatial processing are both regionally and mechanistically distinct.

The frontotemporal theta mechanism for processing temporal information in working memory was not as widely distributed. Instead, we found that the MTL**→**PFC system was the only system that showed preferential processing of an item in time over the ongoing maintenance of item identity, revealing an MTL**→**PFC network that uniquely subserved working memory for both space and time. This finding is consistent with sparse evidence implicating both the MTL and the PFC in temporal memory, sequential ordering, and transitive inference (e.g., if A > B and B > C, then A > C), but the direction of interaction between these regions was not tested previously [37–40]. Importantly, we observed that the MTL directed the PFC during the processing of spatiotemporal information (see [41] for evidence of rodent hippocampal subregion directional connectivity to the PFC for spatial versus temporal processing), while concurrent PFC theta oscillations directed the MTL regardless of the information being processed. These findings suggest that working memory depends on dynamic, bidirectional interactions between the MTL and PFC. We suggest that theta multiplexing provides a plausible mechanism for the domain-general processing of incoming streams of information (e.g., from PFC to MTL) and the domain-specific processing of relevant spatiotemporal information (from MTL to PFC) in working memory.

Finally, the concurrence of MTL**→**PFC and PFC**→**MTL PAC highlights cross-frequency multiplexing as a mechanism for simultaneous, bidirectional MTL–PFC communication. Taken together with spatially distributed patterns of bidirectional MTL-frontal connectivity within the theta band, these findings provide the first demonstration—to our knowledge—of bidirectional MTL–PFC communication in humans. We also observed bidirectional MTL–OFC interactions, but we note that OFC activities were on the net receiving end of MTL theta oscillations during spatial processing and that MTL–OFC interactions did not track working memory for time. Given proposals that the OFC maintains a map of task-related variables analogous to that of the hippocampus [42–43], we suggest that the OFC plays a conjunctive role in distributed MTL theta mechanisms for processing space. In sum, our findings suggest that dynamic, bidirectional communication between the MTL and PFC forms the basis of our everyday cognitive experiences—allowing us to process incoming information in parallel under PFC guidance while also prioritizing information about space and time, dependent on the MTL.

## Materials and methods

### Ethics statement

All subjects gave informed written consent in accordance with the University of California, Berkeley Institutional Review Board (#2010-01-520); Regional Committee for Medical Research Ethics, Region South (#2015/175/REK); or Stanford University Medical Center Institutional Review Board (Protocol ID 11354, IRB Number 4593, panel 5); and also in agreement with the Declaration of Helsinki.

### Subjects

We report data from 10 human adult subjects (mean ± SD [range]: 37 ± 13 [22–69] y; 7 males) who were undergoing intracranial monitoring to localize epileptic foci in preparation for surgical resection. Electrodes were implanted solely on the clinical needs of each patient, and we selected datasets for inclusion via off-site review of individual neuroanatomical coverage. These datasets were collected at one of 3 sites: University of California, Irvine Hospital (7 subjects with stereotactic and/or subdural implants), Oslo University Hospital (2 subjects with stereotactic implants), or Stanford University Medical Center (1 subject with stereotactic and subdural implants).

### Behavioral task

Working memory was tested in a single-trial task paradigm (Fig 1A) [15]. After each 1-s pretrial fixation interval, a starting screen (800-ms duration) indicated whether the upcoming pair of stimuli would be tested for “identity” or spatiotemporal “relation” information. Then, following a 100-ms central fixation interval, 2 common-shape stimuli were presented for 200 ms, each in a specific spatiotemporal configuration (i.e., top/bottom spatial and first/second temporal positions). The test cue was presented (800-ms duration) after a 900- or 1,150-ms delay interval to elicit information-specific selection mechanisms during a second delay interval of the same length. The length of the delays was randomly jittered at 900 or 1,150 ms to preclude anticipatory mechanisms. Then, 2 shapes were presented on the horizontal axis, and subjects responded in a two-alternative forced choice test, resulting in a 0.5 chance rate. In the identity test, subjects indicated whether the pair was the “same” pair they just studied; half of the pairs show 2 old shapes (“yes”), and half of the pairs show 1 old shape and 1 new shape (“no”), using the up and down arrow keys. Subjects indicated which shape had been on the “top” or “bottom” in the spatial relation test and which shape had been presented “first” or “second” in the temporal relation test by using the left and right arrow keys.

The task was fully counterbalanced with 120 trials divided evenly between identity, spatial, and temporal conditions, chosen randomly from a pool of 150 trials with unique stimuli (for a total of 260 stimuli chosen randomly from a pool of 325 unique stimuli). An experimenter went through the experimental task instructions and a set of 6 practice trials with each subject, who was permitted to repeat the practice trials by request. All subjects completed the working memory task (i.e., 120 trials). The task was programmed in E-Prime Professional 2.0 (Psychology Software Tools, Pittsburgh, PA).

Accuracy and correct-trial response time data were submitted to logit and linear mixed-effects models, respectively, with 3 condition-fixed effects and 10 subject-random effects, to confirm that the 3 conditions did not differ in difficulty [44]. We then investigated working memory for space and time by comparing neural activity during the selection of spatial and temporal information, respectively, to the identity control. Electrophysiological data were analyzed for correct trials.

### Electrode localization

Electrodes were localized for each subject based on individual anatomy and then separately transformed into standard MNI space and normalized to the left hemisphere for presentation across subjects (see Fig 1B). Affine point–based registration was used to coregister postimplantation computed tomography (CT) coordinates to the preoperative magnetic resonance (MR) images in BioImage Suite [45]. We determined individual electrode anatomical locations in a group review of each reconstruction under a neurologist (RTK). Subjects were selected based on electrode placement in the MTL (i.e., CA1; CA3/dentate gyrus; subiculum; or parahippocampal, perirhinal, or entorhinal area), lateral PFC (inferior, middle, or superior frontal area), and OFC (orbitofrontal, frontal polar, or medial prefrontal area).

### Data acquisition and preprocessing

Irvine data were acquired using a Nihon Kohden recording system, sampled at 5 or 10 kHz, and resampled offline to 1 kHz. Stanford data were acquired using a Tucker Davis Technologies recording system, sampled at 1.526 kHz, and resampled offline to 1 kHz. Oslo data were acquired using a Nicolet (NicOne) recording system and sampled at 512 Hz. Prior to electrode localization and data preprocessing, the following numbers of electrodes were recorded per subject: S1, 84; S2, 80; S3, 60; S4, 100; S5, 172; S6, 106; S7, 110; S8, 101; S9, 126; and S10, 126.

Raw electrophysiology data traces were manually inspected under the supervision of a neurologist (RTK), who was blinded to electrode locations and experimental task parameters. Channels and epochs displaying epileptiform activity or artifactual signal (from poor contact, machine noise, etc.) as well as those placed on tissue that was later resected were marked for exclusion. Remaining channels were filtered with 1-Hz high-pass and 200-Hz low-pass (165 Hz for Oslo data) finite impulse response filters and demeaned, and 60-Hz line noise harmonics (50 Hz for Oslo data) were removed using discrete Fourier transform. The filtered data we re-inspected to mark any channels or epochs containing residual artifacts for exclusion. Then, every artifact-free electrode within the MTL, PFC, or OFC was rereferenced to the next adjacent electrode—spaced at 5 or 10 mm within that region—using bipolar montages to create virtual electrodes with minimized volume conduction [46–47]. The final dataset contained 254 virtual electrodes (mean ± SD [range]) per subject: 4 ± 2 (1–7) MTL, 12 ± 8 (4–28 in 9 out of 10 subjects) PFC, 10 ± 14 (1–47 in 9 out of 10 subjects) OFC (see Fig 1B).

We then epoched the continuous data into trials with 1-s buffers, excluded trials overlapping with epochs that had been marked during inspection, and manually re-inspected the data to reject any trials with residual noise. The final dataset included an average of 100 correct trials per subject (mean ± SD [range]): 33 ± 3 (26–37) identity, 34 ± 4 (28–39) spatial relation, 33 ± 3 (28–37) temporal relation. There were too few incorrect trials for meaningful analysis: 10 ± 8 (0–25) per subject. Finally, the data were epoched into 3 segments per trial for analysis (see Fig 1A): (1) 200-ms pretrial baseline interval extending from 250 to 50 ms before the start screen; (2) 1,500-ms encoding and precue delay interval extending from the onset of the first stimulus; and (3) 900-ms postcue delay interval extending from the offset of the test cue. Preprocessing routines were performed using the FieldTrip [48] and EEGLAB [49] toolboxes for MATLAB (MathWorks Inc., Natick, MA).

### ERPs

The correct-trial 200-ms pretrial baseline, 1,500-ms encoding and precue delay, and 900-ms postcue delay data segments were zero padded to 7,500 ms to minimize filtering-induced edge artifacts and passed through a 30-Hz low-pass finite impulse response filter [15]. Task-induced ERPs were computed over the encoding and delay intervals by absolute baseline-correcting the outputs on the temporal mean of the pretrial baseline.

Task-induced ERPs were tested for condition differences between identity and relation trials over the encoding and precue delay interval as well as between identity, spatial, and temporal trials over the postcue delay. Within-subject statistical testing employed a Monte Carlo method with cluster-based maximum correction for multiple comparisons [26]. An independent-samples *t* test (or F-test for 3 conditions) was used to identify clusters of contiguous data points showing a difference between conditions—thresholded at 0.05, two-tailed—and then the *t* statistics (or F-statistics) were summed over all data points per cluster to calculate cluster size. Effects were clustered per electrode based on temporal adjacency. Then, condition labels were randomly shuffled, and the same clustering procedure was applied; this procedure was repeated 1,000 times to create a normal distribution of null effects. Observed clusters were considered significant if fewer than 5% of randomizations yielded a larger effect (i.e., cluster-corrected α = 0.05). Statistical testing was performed using FieldTrip [48] functions in MATLAB (MathWorks, Natick, MA).

### Spectral decomposition

Time–frequency representations of power were computed on the correct-trial 200-ms pretrial baseline, 1,500-ms encoding and precue delay, and 900-ms postcue delay data segments. Data segments were zero padded to 7,500 ms and passed through 24 logarithmically spaced bandpass finite impulse response filters between 1 and 192 Hz (162 Hz for Oslo data). The Hilbert transform was used to extract the analytic amplitude envelope from each filtered time series, which was squared to produce raw power values.

Task-induced power was analyzed per trial using a statistical bootstrapping procedure. Baseline power values were pooled into a single time series for each channel and frequency, from which we randomly selected and averaged r data points (r = number of trials in that subject’s dataset). This step was repeated 1,000 times to create normal distributions of electrode and frequency-resolved pretrial baseline data. Encoding and delay raw power data were z-scored on the pretrial baseline distributions to assess the significance of task-induced effects (for a similar approach, see [15]).

Power outputs were then down-sampled to 50-ms temporal resolution and submitted to within-subject statistical testing. Cluster-based permutation testing of task-induced power was equivalent to that for ERPs, with clustering on the time and frequency dimensions. Across all 254 electrodes, 8 (5 PFC plus 3 OFC across 4 out of 10 subjects) showed condition differences in power at any time–frequency cluster during encoding or delay. These 8 electrodes were excluded from further analysis.

### PLV

Interelectrode phase synchrony was computed on the correct-trial 900-ms delay data segments between signals across each electrode pair within the same hemisphere (e.g., left MTL–MTL, MTL–PFC, MTL–OFC). First, the trial-wise mean for correct-trial data segments was subtracted from each correct-trial data segment to minimize contamination from simultaneous voltage changes on phase estimates [13,15,31]. Then, complex time–frequency representations were computed using the Hilbert bandpass decomposition procedure with the same parameters described above, from 2 to 20 Hz. PLV was calculated at each timepoint across all correct trials from the imaginary part of the Hilbert-transformed complex signal [28–29].

### PSI

Theta band directional connectivity was computed on the correct-trial 900-ms delay data segments between signals across each interregional electrode pair within the same hemisphere (e.g., left MTL–PFC, MTL–OFC). First, the trial-wise mean for correct-trial data segments was subtracted from each correct-trial data segment [13,15,31]. The data segments were zero padded to 4 s, and spectral representations were computed using an adaptive, frequency-dependent sliding time window of 3 cycles’ length (Δt = 3/f) for frequencies between 3 and 7 Hz (1-Hz steps, 2-Hz bandwidth) (for a similar approach, see [15,50]). Data were multiplied with a Hanning taper, which reduces spectral leakage and allows us to keep the bandwidth constant for computation of PSI [21,30–31]. Cross-spectral density was calculated between the complex Fourier outputs, from which PSI was computed [30].

Per-subject statistical analysis of PSI was performed for the delay intervals by standardizing the outputs against a surrogate distribution via bootstrapping. At each electrode pair and frequency point, we randomly shuffled the frequencies in one signal and recomputed PSI. This step was repeated 1,000 times to create normal distributions of electrode pair and frequency-resolved null PSI data. Raw PSI outputs were z-scored on the null distributions to correct for any spurious results and assess the significance of directional effects (for a similar approach, see [15,31]). MTL leads were defined as PSI z > 1.96 and frontal leads as z < −1.96 (i.e., α = 0.05).

All PSI outputs were then submitted to group statistical testing to confirm that directionality shifted from pre- to postcue delay. MTL–PFC and MTL–OFC outputs were submitted separately to linear mixed-effects models, with 2 task interval–fixed effects and subject- and electrode pair–random effects. All subjects and electrode pairs were included: *N* subjects (*n* electrode pairs) = 9 (172) MTL–PFC, 7 (303) MTL–OFC.

### Cross-spectral directionality

Cross-spectral directionality was computed on the correct-trial 900-ms delay data segments between signals within each electrode (i.e., MTL–MTL, PFC–PFC, OFC–OFC) and across each interregional electrode pair within the same hemisphere (e.g., left MTL–PFC, PFC–MTL, MTL–OFC, OFC–MTL). Theta band inputs were obtained using the same procedure as described above in the analysis of PSI. Separately, time–frequency representations of amplitude were computed using the Hilbert bandpass procedure with the same parameters described in the analysis of power at all higher frequencies: 9.5 to 152.5 Hz (128 Hz for Oslo data). Then, each amplitude-envelope time series output was submitted to the theta range spectral decomposition procedure, which produced theta band complex Fourier outputs of each higher-frequency amplitude. Cross-spectral density was calculated between the complex Fourier outputs of actual theta and each amplitude frequency range, from which PSI was computed [21].

Per-subject statistical analysis of cross-spectral PSI was again performed via bootstrapping, as in the analysis of theta band PSI. The z-scored outputs were then averaged across frequencies to index mean directionality. This procedure identified electrodes (for electrodes within each region) and electrode pairs (for pairs of electrodes between the MTL and PFC/OFC) in which theta phase directed higher-frequency amplitudes during the postcue delay. Phase directionality was defined as PSI z > 1.96 (i.e., α = 0.05).

All cross-spectral directionality outputs were then submitted to group statistical testing to confirm that directionality shifted from pre- to postcue delay. For each region (i.e., MTL, PFC, OFC) and interregional network (MTL**→**PFC, PFC**→**MTL, MTL**→**OFC, OFC**→**MTL), outputs were submitted to a linear mixed-effects model with 2 task interval–fixed effects and subject- and electrode/electrode pair–random effects. All subjects and electrodes/electrode pairs were included: *N* subjects (*n* electrodes/electrode pairs) = 10 (36) MTL, 9 (111) PFC, 8 (99) OFC, 9 (172) MTL–PFC, and 7 (303) MTL–OFC. The individual electrode pairs that were significantly phase-directing in the MTL**→**PFC/OFC and PFC/OFC→MTL directions were then submitted to another set of models to test whether directionality was greater at certain amplitude frequencies and/or in one direction. These models comprised 16 amplitude frequency (9.5–128 Hz) and 2 direction-fixed effects, with subject- and electrode pair–random effects. All subjects with theta phase–directing electrodes in each region were included: 8 (13) MTL, 8 (52) PFC, 8 (38) OFC, 9 (56) MTL→PFC, 9 (85) PFC→MTL, 5 (93) MTL**→**OFC, and 5 (115) OFC→MTL.

### PAC

Time–frequency representations of individual theta PAC were computed per condition on the 900-ms postcue delay data segments for each theta-phase directing electrode and directional electrode pair identified in the cross-spectral directionality analysis. First, peak theta band range was estimated on an individual basis from the task-induced power data over the encoding and precue interval, cf. [50]. We selected task-induced power in the 6 frequency ranges bandpass-filtered between 1.5 and 10 Hz and then averaged over the trial, electrode, and time dimensions to reveal individual maxima. Peaks were detected across the theta band: *n =* 3 at 2 to 5 Hz, *n =* 2 at 3 to 6 Hz, *n =* 2 at 4 to 7 Hz, and *n =* 3 at 5 to 8 Hz. We visually inspected the task-induced power data without averaging to confirm that these peak frequencies exhibited comparable responses across subjects.

Then, the trial-wise mean for correct-trial data segments was subtracted from each correct-trial data segment separately for the identity, spatial, and temporal conditions [13,15,31]. The individual theta-phase time series and time–frequency representations of amplitudes centered at 9.5 to 152.5 Hz (128 Hz for Oslo data) were computed from the outputs using the Hilbert bandpass procedure with the same parameters described above. PAC was computed from the outputs at each time–frequency point using circular statistics across all correct trials per condition [23]. For each timepoint and amplitude frequency, circular–linear Pearson correlation [51] was applied between vectors of r theta phase data points and r higher-frequency amplitude data points (r = number of trials in that condition). This procedure outputs time–frequency representations of raw PAC for each electrode/electrode pair.

Per-subject statistical analysis of PAC was performed by standardizing the outputs against surrogate distributions via bootstrapping [23,27,31]. At each electrode/electrode pair and time–frequency point, we randomly shuffled the amplitude frequencies across trials and recomputed PAC. This step was repeated 100 times to create normal distributions of electrode/electrode pair and time–frequency–resolved null PAC data. Raw PAC outputs were z-scored on the null distributions to correct for any spurious results and assess the significance of PAC effects.

PAC outputs were then down-sampled to 50-ms temporal resolution and submitted to group statistical testing of condition differences. For each region and interregional, directional system, all PAC outputs were submitted to 2 linear mixed-effects models, with 2 condition-, 16 amplitude frequency–(9.5–128 Hz), and 19 time-fixed effects, as well as subject- and electrode/electrode pair–random effects. Spatial and temporal PAC were tested separately against the identity control. We additionally tested PAC for strength of directional coupling by submitting the MTL→PFC/OFC and PFC/OFC→MTL PAC data to models with a fourth fixed effect: direction. As in the cross-spectral directionality models, all subjects with theta-phase directing electrodes in each region were included. Effects were considered significant if they passed the Bonferroni-corrected threshold for multiple comparisons (i.e., Bonferroni-corrected α = 0.05/m, with m = number of main + interaction fixed effects [52]).

## Supporting information

S1 Fig Task-induced ERPs over encoding and delay by condition. (A) Task-induced ERPs over encoding, precue, and postcue intervals in a representative MTL electrode (cf. Fig 2). No condition differences were observed. Black, identity trials; blue, spatiotemporal relation trials (encoding and precue delay) or spatial trials (postcue delay); teal, temporal trials (postcue delay); shaded area, SEM. (B) Equivalent to panel A: PFC. (C) Equivalent to panel A: OFC. Underlying data can be found in University of California, Berkeley, Collaborative Research in Computational Neuroscience database (http://dx.doi.org/10.6080/K0VX0DQD). ERP, event-related potential; MTL, medial temporal lobe; OFC, orbitofrontal cortex; PFC, prefrontal cortex.
(TIF)

S2 Fig Frontal theta PAC dynamics during the postcue delay. (A) PAC by condition during the postcue delay in a representative PFC electrode (cf. Fig 2). The PFC showed variable, transient PAC across the spectrum of amplitude envelopes (z > 1.96, *p <* 0.05). No condition differences were observed. The black block indicates the amplitude data range depicted in panel B. (B) The distribution of raw higher-frequency amplitudes across 18 theta phase bins, by condition, normalized by the maximum amplitude across all of the phase bins. AMP frequency range: high-frequency broadband (centered at 90.5 Hz). Gray, identity trials; blue, spatial trials; teal, temporal trials. (C) Equivalent to panel A: OFC. (D) Equivalent to panel B: OFC. Underlying data can be found in University of California, Berkeley, Collaborative Research in Computational Neuroscience database (http://dx.doi.org/10.6080/K0VX0DQD). AMP, amplitude; DIFF, difference (i.e., spatial–identity, temporal–identity); FREQ, frequency; NORM, normalized; OFC, orbitofrontal cortex; PAC, phase-amplitude coupling; PFC, prefrontal cortex.
(TIF)

S1 Table MTL–PFC and MTL–OFC cross-spectral directionality group model results. DF, degrees of freedom; FREQ, amplitude frequency; MTL, medial temporal lobe; OFC, orbitofrontal cortex; PFC, prefrontal cortex.
(DOCX)

S2 Table Local theta PAC group model results by condition and region. ** = significant effect; bold = result of interest. DF, degrees of freedom; FREQ, amplitude frequency; PAC, phase-amplitude coupling.
(DOCX)

S3 Table MTL–PFC theta PAC group model results by condition and direction. ** = significant effect; bold = result of interest. DF, degrees of freedom; FREQ, amplitude frequency; MTL, medial temporal lobe; PAC, phase-amplitude coupling; PFC, prefrontal cortex.
(DOCX)

S4 Table MTL–OFC theta PAC group model results by condition and direction. ** = significant effect; bold = result of interest. DF, degrees of freedom; FREQ, amplitude frequency; MTL, medial temporal lobe; OFC, orbitofrontal cortex; PAC, phase-amplitude coupling.
(DOCX)

## Abbreviations

CT: computed tomography
ERP: event-related potential
MTL: medial temporal lobe
MR: magnetic resonance
OFC: orbitofrontal cortex
PAC: phase-amplitude coupling
PFC: prefrontal cortex
PLV: phase-locking value
PSI: Phase Slope Index
RAD: radians
