# Supplementary figures and images for: Dynamic frontotemporal systems process space and time in working memory
Source: PLoS Biol. 2018 Mar 30;16(3):e2004274. doi: 10.1371/journal.pbio.2004274 (PMC5895055; doi:10.1371/journal.pbio.2004274)

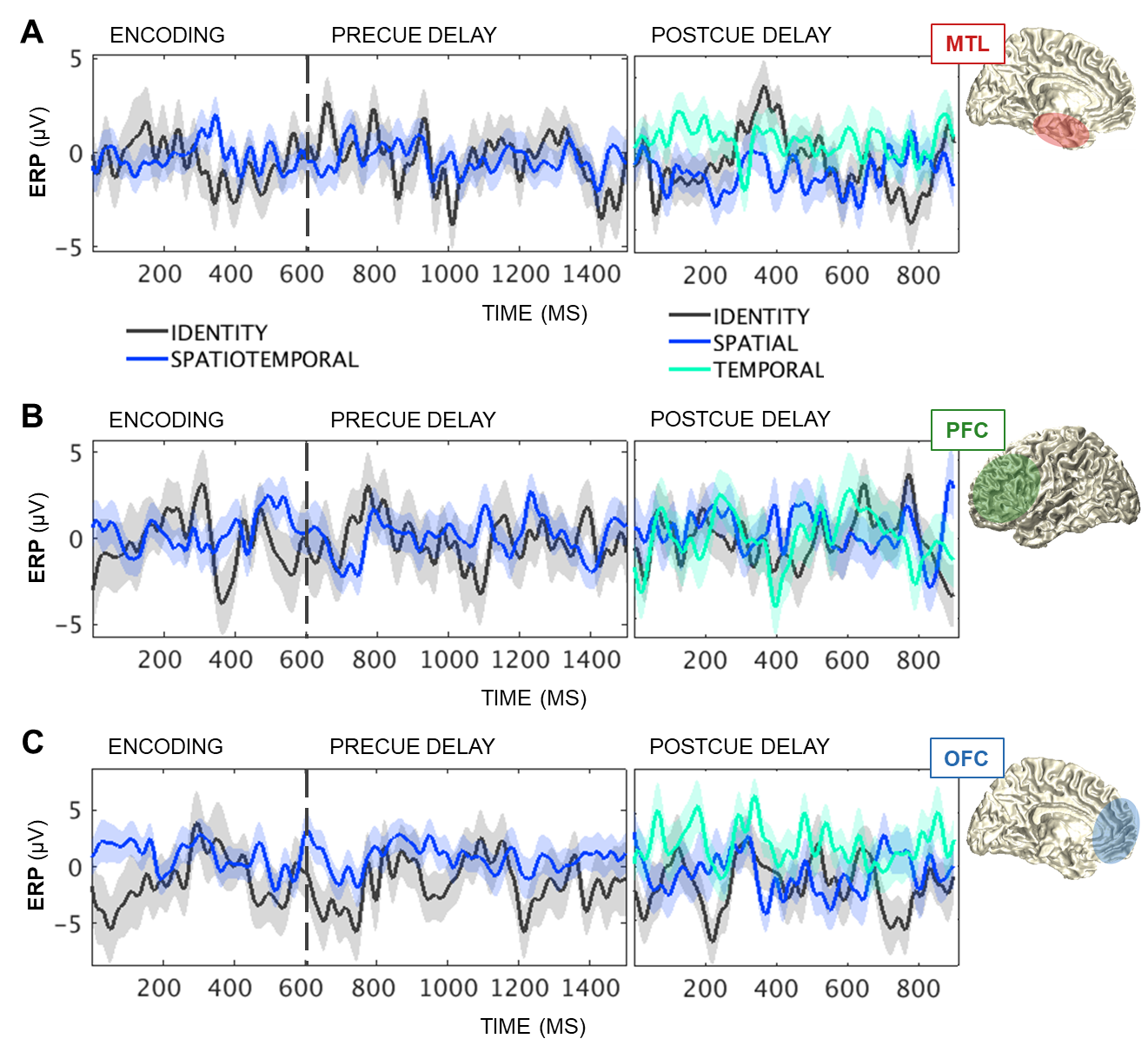

Supplement: S1 Fig — (A) Task-induced ERPs over encoding, precue, and postcue intervals in a representative MTL electrode (cf. Fig 2). No condition differences were observed. Black, identity trials; blue, spatiotemporal relation trials (encoding and precue delay) or spatial trials (postcue delay); teal, temporal trials (postcue delay); shaded area, SEM. (B) Equivalent to panel A: PFC. (C) Equivalent to panel A: OFC. Underlying data can be found in University of California, Berkeley, Collaborative Research in Computational Neuroscience database (http://dx.doi.org/10.6080/K0VX0DQD). ERP, event-related potential; MTL, medial temporal lobe; OFC, orbitofrontal cortex; PFC, prefrontal cortex. (TIF) [file pbio.2004274.s001.tif]

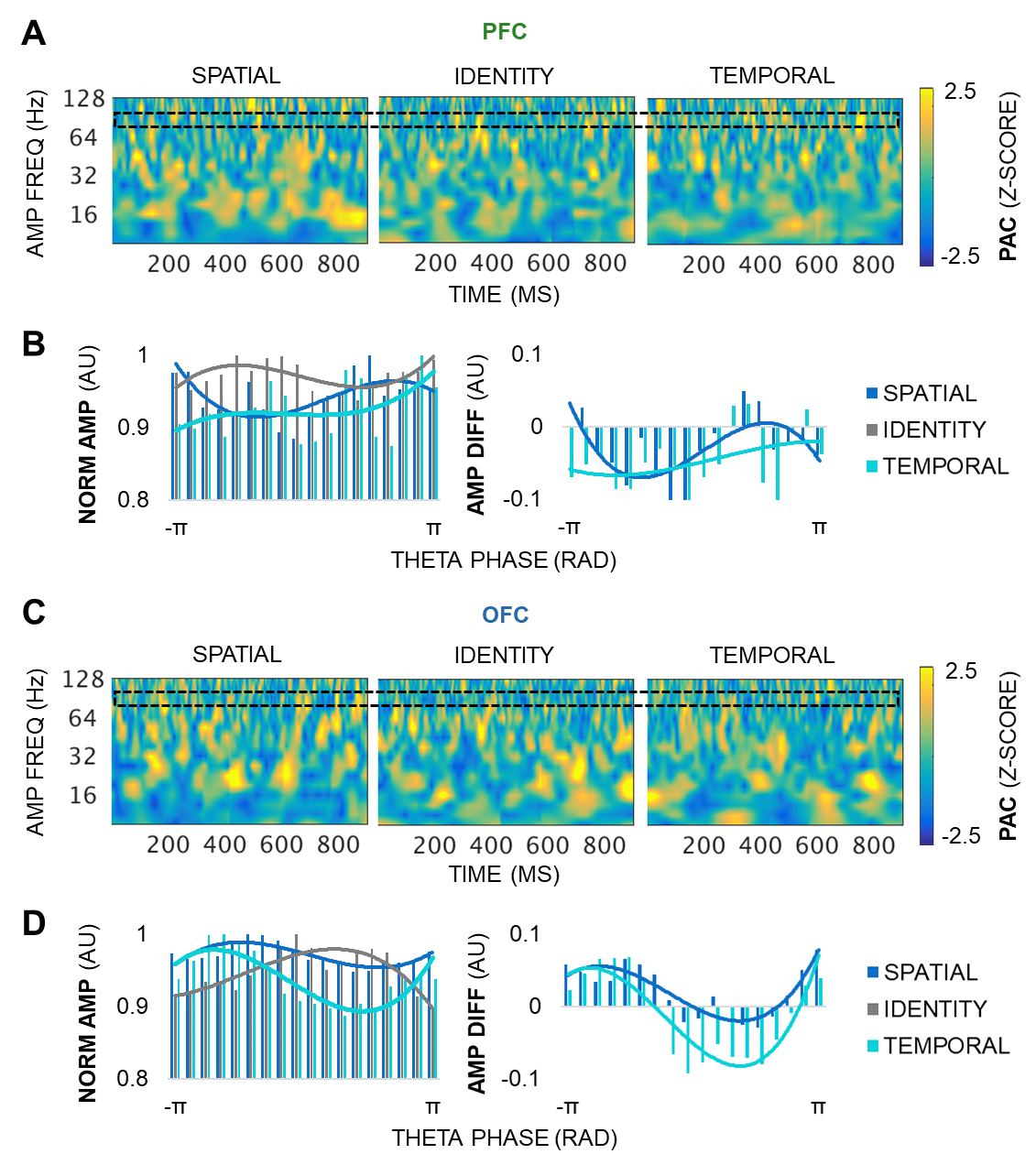

Supplement: S2 Fig — (A) PAC by condition during the postcue delay in a representative PFC electrode (cf. Fig 2). The PFC showed variable, transient PAC across the spectrum of amplitude envelopes (z > 1.96, p < 0.05). No condition differences were observed. The black block indicates the amplitude data range depicted in panel B. (B) The distribution of raw higher-frequency amplitudes across 18 theta phase bins, by condition, normalized by the maximum amplitude across all of the phase bins. AMP frequency range: high-frequency broadband (centered at 90.5 Hz). Gray, identity trials; blue, spatial trials; teal, temporal trials. (C) Equivalent to panel A: OFC. (D) Equivalent to panel B: OFC. Underlying data can be found in University of California, Berkeley, Collaborative Research in Computational Neuroscience database (http://dx.doi.org/10.6080/K0VX0DQD). AMP, amplitude; DIFF, difference (i.e., spatial–identity, temporal–identity); FREQ, frequency; NORM, normalized; OFC, orbitofrontal cortex; PAC, phase-amplitude coupling; PFC, prefrontal cortex. (TIF) [file pbio.2004274.s002.tif]
