# Supplementary material for: Dynamic frontotemporal systems process space and time in working memory
Source: PLoS Biol. 2018 Mar 30;16(3):e2004274. doi: 10.1371/journal.pbio.2004274 (PMC5895055; doi:10.1371/journal.pbio.2004274)
Supplement: S1 Table — DF, degrees of freedom; FREQ, amplitude frequency; MTL, medial temporal lobe; OFC, orbitofrontal cortex; PFC, prefrontal cortex. (DOCX) [file pbio.2004274.s003.docx]

**S1 Table**

**MTL-PFC and MTL-OFC cross-spectral directionality group model results**

| **MTL-PFC** | F-Statistic | DF | Cohen’s d | P-Value |
| --- | --- | --- | --- | --- |
| FREQ | 2.23 | 1,2252 | 0.18 | 0.14 |
| DIRECTION | 0.00 | 1,2252 | 0.01 | 0.96 |
| FREQ×DIRECTION | 2.46 | 1,2252 | 0.19 | 0.12 |

| **MTL-OFC** | F-Statistic | DF | Cohen’s d | P-Value |
| --- | --- | --- | --- | --- |
| FREQ | 0.00 | 1,3324 | 0.00 | 0.99 |
| DIRECTION | 0.07 | 1,3324 | 0.03 | 0.79 |
| FREQ×DIRECTION | 0.05 | 1,3324 | 0.02 | 0.83 |

FREQ, amplitude frequency; DF, degrees of freedom.
