# Supplementary material for: Dynamic frontotemporal systems process space and time in working memory
Source: PLoS Biol. 2018 Mar 30;16(3):e2004274. doi: 10.1371/journal.pbio.2004274 (PMC5895055; doi:10.1371/journal.pbio.2004274)
Supplement: S2 Table — ** = significant effect; bold = result of interest. DF, degrees of freedom; FREQ, amplitude frequency; PAC, phase-amplitude coupling. (DOCX) [file pbio.2004274.s004.docx]

**S2 Table**

**Local theta PAC group model results by condition and region**

| **MTL** |  | SPATIAL VS. IDENTITY | | TEMPORAL VS. IDENTITY | |
| --- | --- | --- | --- | --- | --- |
|  | DF | F-Statistic | Cohen’s d | F-Statistic | Cohen’s d |
| **CONDITION** | **1,7896** | **19.85** | **1.82**** | 0.33 | 0.23 |
| FREQ | 1,7896 | 12.03 | 1.42** | 1.20 | 0.45 |
| TIME | 1,7896 | 1.84 | 0.55 | 2.07 | 0.59 |
| **CONDITION×FREQ** | **1,7896** | **17.85** | **1.73**** | 0.25 | 0.21 |
| CONDITION×TIME | 1,7896 | 5.99 | 1.00 | 3.92 | 0.81 |
| FREQ×TIME | 1,7896 | 0.66 | 0.33 | 1.49 | 0.50 |
| CONDITION×FREQ×TIME | 1,7896 | 3.46 | 0.76 | 1.87 | 0.56 |

| **PFC** |  | SPATIAL VS. IDENTITY | | TEMPORAL VS. IDENTITY | |
| --- | --- | --- | --- | --- | --- |
|  | DF | F-Statistic | Cohen’s d | F-Statistic | Cohen’s d |
| CONDITION | 1,31608 | 1.82 | 0.27 | 1.54 | 0.25 |
| FREQ | 1,31608 | 0.03 | 0.04 | 0.01 | 0.02 |
| TIME | 1,31608 | 1.55 | 0.25 | 3.85 | 0.39 |
| CONDITION×FREQ | 1,31608 | 0.00 | 0.00 | 0.20 | 0.09 |
| CONDITION×TIME | 1,31608 | 0.06 | 0.05 | 1.98 | 0.28 |
| FREQ×TIME | 1,31608 | 2.51 | 0.31 | 2.25 | 0.30 |
| CONDITION×FREQ×TIME | 1,31608 | 1.03 | 0.20 | 0.81 | 0.18 |

| **OFC** |  | SPATIAL VS. IDENTITY | | TEMPORAL VS. IDENTITY | |
| --- | --- | --- | --- | --- | --- |
|  | DF | F-Statistic | Cohen’s d | F-Statistic | Cohen’s d |
| CONDITION | 1,23096 | 0.22 | 0.11 | 1.36 | 0.27 |
| FREQ | 1,23096 | 0.00 | 0.01 | 0.37 | 0.14 |
| TIME | 1,23096 | 0.10 | 0.07 | 1.92 | 0.32 |
| CONDITION×FREQ | 1,23096 | 0.10 | 0.07 | 0.35 | 0.14 |
| CONDITION×TIME | 1,23096 | 4.29 | 0.48 | 0.34 | 0.14 |
| FREQ×TIME | 1,23096 | 0.01 | 0.03 | 2.03 | 0.33 |
| CONDITION×FREQ×TIME | 1,23096 | 2.44 | 0.36 | 0.68 | 0.19 |

**, significant effect; bold, result of interest; FREQ, amplitude frequency; DF, degrees of freedom.
