# Supplementary material for: Dynamic frontotemporal systems process space and time in working memory
Source: PLoS Biol. 2018 Mar 30;16(3):e2004274. doi: 10.1371/journal.pbio.2004274 (PMC5895055; doi:10.1371/journal.pbio.2004274)
Supplement: S3 Table — ** = significant effect; bold = result of interest. DF, degrees of freedom; FREQ, amplitude frequency; MTL, medial temporal lobe; PAC, phase-amplitude coupling; PFC, prefrontal cortex. (DOCX) [file pbio.2004274.s005.docx]

**S3 Table**

**MTL-PFC theta PAC group model results by condition and direction**

| **MTL**🡪**PFC** |  | SPATIAL VS. IDENTITY | | TEMPORAL VS. IDENTITY | |
| --- | --- | --- | --- | --- | --- |
|  | DF | F-Statistic | Cohen’s d | F-Statistic | Cohen’s d |
| **CONDITION** | **1,34040** | **41.53** | **1.23**** | **15.35** | **0.75**** |
| FREQ | 1,34040 | 17.17 | 0.79** | 7.82 | 0.53** |
| TIME | 1,34040 | 37.17 | 1.16** | 10.38 | 0.61** |
| **CONDITION×FREQ** | **1,34040** | **15.00** | **0.74**** | 3.00 | 0.33 |
| **CONDITION×TIME** | **1,34040** | **36.37** | **1.15**** | 2.16 | 0.28 |
| FREQ×TIME | 1,34040 | 10.67 | 0.62** | 0.40 | 0.12 |
| **CONDITION×FREQ×TIME** | **1,34040** | **12.53** | **0.68**** | 0.40 | 0.12 |

| **PFC**🡪**MTL** |  | SPATIAL VS. IDENTITY | | TEMPORAL VS. IDENTITY | |
| --- | --- | --- | --- | --- | --- |
|  | DF | F-Statistic | Cohen’s d | F-Statistic | Cohen’s d |
| CONDITION | 1,51672 | 1.04 | 0.16 | 4.64 | 0.33 |
| FREQ | 1,51672 | 5.76 | 0.37 | 8.36 | 0.45** |
| TIME | 1,51672 | 2.51 | 0.25 | 1.73 | 0.20 |
| CONDITION×FREQ | 1,51672 | 2.98 | 0.27 | 6.17 | 0.38 |
| CONDITION×TIME | 1,51672 | 1.54 | 0.19 | 0.65 | 0.12 |
| FREQ×TIME | 1,51672 | 4.34 | 0.32 | 3.55 | 0.29 |
| CONDITION×FREQ×TIME | 1,51672 | 3.47 | 0.29 | 2.37 | 0.24 |

| **MTL-PFC** |  | SPATIAL VS. IDENTITY | | TEMPORAL VS. IDENTITY | |
| --- | --- | --- | --- | --- | --- |
|  | DF | F-Statistic | Cohen’s d | F-Statistic | Cohen’s d |
| CONDITION | 1,85712 | 39.49 | 0.75** | 19.45 | 0.53** |
| FREQ | 1,85712 | 22.11 | 0.56** | 13.40 | 0.44** |
| TIME | 1,85712 | 38.20 | 0.74** | 11.93 | 0.41** |
| **DIRECTION** | **1,85712** | **46.10** | **0.81**** | **36.92** | **0.73**** |
| CONDITION×FREQ | 1,85712 | 17.64 | 0.50** | 6.40 | 0.30 |
| CONDITION×TIME | 1,85712 | 35.89 | 0.72** | 2.73 | 0.20 |
| **CONDITION×DIRECTION** | **1,85712** | **31.24** | **0.67**** | **19.17** | **0.52**** |
| FREQ×TIME | 1,85712 | 14.28 | 0.45** | 1.67 | 0.16 |
| FREQ×DIRECTION | 1,85712 | 22.12 | 0.56** | 15.88 | 0.48** |
| TIME×DIRECTION | 1,85712 | 32.29 | 0.68** | 10.97 | 0.40** |
| CONDITION×FREQ×TIME | 1,85712 | 15.57 | 0.47** | 0.00 | 0.00 |
| **CONDITION×FREQ×DIRECTION** | **1,85712** | **16.54** | **0.49**** | 8.45 | 0.35 |
| **CONDITION×TIME×DIRECTION** | **1,85712** | **29.32** | **0.65**** | 2.69 | 0.20 |
| FREQ×TIME×DIRECTION | 1,85712 | 14.65 | 0.46** | 2.83 | 0.20 |
| **CONDITION×FREQ×TIME×DIRECTION** | **1,85712** | **15.20** | **0.47**** | 0.24 | 0.06 |

**, significant effect; bold, result of interest; FREQ, amplitude frequency; DF, degrees of freedom.
