# Supplementary material for: Dynamic frontotemporal systems process space and time in working memory
Source: PLoS Biol. 2018 Mar 30;16(3):e2004274. doi: 10.1371/journal.pbio.2004274 (PMC5895055; doi:10.1371/journal.pbio.2004274)
Supplement: S4 Table — ** = significant effect; bold = result of interest. DF, degrees of freedom; FREQ, amplitude frequency; MTL, medial temporal lobe; OFC, orbitofrontal cortex; PAC, phase-amplitude coupling. (DOCX) [file pbio.2004274.s006.docx]

**S4 Table**

**MTL-OFC theta PAC group model results by condition and direction**

| **MTL**🡪**OFC** |  | SPATIAL VS. IDENTITY | | TEMPORAL VS. IDENTITY | |
| --- | --- | --- | --- | --- | --- |
|  | DF | F-Statistic | Cohen’s d | F-Statistic | Cohen’s d |
| **CONDITION** | **1,56536** | **21.33** | **0.68**** | 0.06 | 0.04 |
| FREQ | 1,56536 | 6.22 | 0.37 | 0.18 | 0.06 |
| TIME | 1,56536 | 9.91 | 0.46** | 5.58 | 0.35 |
| **CONDITION×FREQ** | **1,56536** | **8.04** | **0.42**** | 0.20 | 0.07 |
| CONDITION×TIME | 1,56536 | 5.84 | 0.36 | 1.33 | 0.17 |
| FREQ×TIME | 1,56536 | 4.86 | 0.33 | 1.52 | 0.18 |
| CONDITION×FREQ×TIME | 1,56536 | 3.20 | 0.26 | 0.06 | 0.04 |

| **OFC**🡪**MTL** |  | SPATIAL VS. IDENTITY | | TEMPORAL VS. IDENTITY | |
| --- | --- | --- | --- | --- | --- |
|  | DF | F-Statistic | Cohen’s d | F-Statistic | Cohen’s d |
| CONDITION | 1,69912 | 0.15 | 0.05 | 1.67 | 0.17 |
| FREQ | 1,69912 | 0.90 | 0.13 | 0.19 | 0.06 |
| TIME | 1,69912 | 1.51 | 0.16 | 0.75 | 0.12 |
| CONDITION×FREQ | 1,69912 | 1.24 | 0.15 | 0.09 | 0.04 |
| CONDITION×TIME | 1,69912 | 2.88 | 0.23 | 2.62 | 0.21 |
| FREQ×TIME | 1,69912 | 1.89 | 0.18 | 0.01 | 0.01 |
| CONDITION×FREQ×TIME | 1,69912 | 2.27 | 0.20 | 0.62 | 0.10 |

| **MTL-OFC** |  | SPATIAL VS. IDENTITY | | TEMPORAL VS. IDENTITY | |
| --- | --- | --- | --- | --- | --- |
|  | DF | F-Statistic | Cohen’s d | F-Statistic | Cohen’s d |
| CONDITION | 1,126448 | 19.12 | 0.43** | 0.09 | 0.03 |
| FREQ | 1,126448 | 7.10 | 0.26 | 0.32 | 0.06 |
| TIME | 1,126448 | 11.41 | 0.33** | 3.23 | 0.18 |
| **DIRECTION** | **1,126448** | **10.18** | **0.31**** | 0.20 | 0.04 |
| CONDITION×FREQ | 1,126448 | 9.27 | 0.30** | 0.08 | 0.03 |
| CONDITION×TIME | 1,126448 | 8.41 | 0.29 | 0.15 | 0.04 |
| **CONDITION×DIRECTION** | **1,126448** | **13.64** | **0.36**** | 0.46 | 0.07 |
| FREQ×TIME | 1,126448 | 6.63 | 0.25 | 1.20 | 0.11 |
| FREQ×DIRECTION | 1,126448 | 6.20 | 0.25 | 0.37 | 0.06 |
| TIME×DIRECTION | 1,126448 | 10.02 | 0.31** | 1.38 | 0.12 |
| CONDITION×FREQ×TIME | 1,126448 | 5.06 | 0.22 | 0.01 | 0.01 |
| CONDITION×FREQ×DIRECTION | 1,126448 | 8.15 | 0.28 | 0.02 | 0.01 |
| CONDITION×TIME×DIRECTION | 1,126448 | 8.60 | 0.29 | 0.05 | 0.02 |
| FREQ×TIME×DIRECTION | 1,126448 | 6.55 | 0.25 | 0.76 | 0.09 |
| CONDITION×FREQ×TIME×DIRECTION | 1,126448 | 5.47 | 0.23 | 0.12 | 0.03 |

**, significant effect; bold, result of interest; FREQ, amplitude frequency; DF, degrees of freedom.
